# Synthesis of tetrazoles catalyzed by a new and recoverable nanocatalyst of cobalt on modified boehmite NPs with 1,3-bis(pyridin-3-ylmethyl)thiourea†

**DOI:** 10.1039/d2ra07510e

**Published:** 2023-03-17

**Authors:** Arida Jabbari, Parisa Moradi, Bahman Tahmasbi

**Affiliations:** a Department of Chemistry, Qeshm Branch, Islamic Azad University Qeshm Iran arida_jabbari@yahoo.com; b Department of Chemistry, Faculty of Science, Ilam University P.O. Box 69315516 Ilam Iran

## Abstract

In the first part of this work, boehmite nanoparticles (BNPs) were synthesized from aqueous solutions of NaOH and Al(NO_3_)_3_·9H_2_O. Then, the BNPs surface was modified using 3-choloropropyltrimtoxysilane (CPTMS) and then 1,3-bis(pyridin-3-ylmethyl)thiourea ((PYT)_2_) was anchored on the surface of the modified BNPs (CPTMS@BNPs). In the final step, a complex of cobalt was stabilized on its surface (Co-(PYT)_2_@BNPs). The final obtained nanoparticles were characterized by FT-IR spectra, TGA analysis, SEM imaging, WDX analysis, EDS analysis, and XRD patterns. In the second part, Co-(PYT)_2_@BNPs were used as a highly efficient, retrievable, stable, and organic–inorganic hybrid nanocatalyst for the formation of organic heterocyclic compounds such as tetrazole derivatives. Co-(PYT)_2_@BNPs as a novel nanocatalyst are stable and have a heterogeneous nature; therefore, they can be recovered and reused again for several consecutive runs without any re-activation.

## Abstract 2

A new complex of cobalt was stabilized on boehmite nanoparticles as an efficient and selective nanocatalyst for the synthesis of tetrazoles. This catalyst is stable and it has heterogeneity nature; therefore, it can reused for several runs.

## Graphical abstract

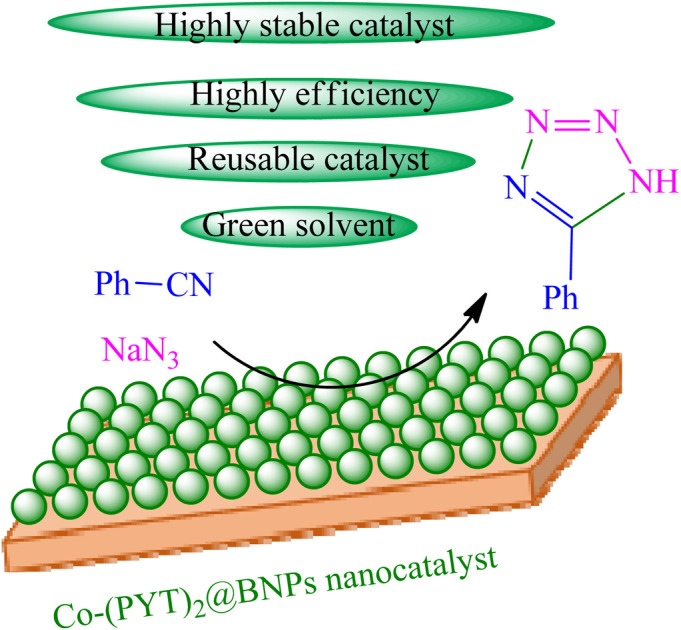

## 1 Introduction

In recent years, boehmite nanoparticles (BNPs) have attracted interest from both practical and fundamental viewpoints.^1,2^ In fact, boehmite is aluminum oxyhydroxide (γ-AlOOH) and it is the most stable phase of alumina after gibbsite.^3–6^ Boehmite consists of double sheets of oxygen octahedron with Al-atoms at their centers.^7–10^ The boehmite sheets themselves are composed of octahedral chains with a cubic orthorhombic unit cell.^2,11^ Also, BNPs are very stable and they are not moisture or air sensitive.^12,13^ Therefore, BNPs can synthesized in aqueous media without inert atmosphere by available materials such as inexpensive aluminum salts.^14^ The physical and chemical properties of boehmite are strongly dependent on the experimental condition of its synthesis.^13^ For example, BNPs were synthesized by different methods such as hydrolysis of aluminum salts,^2^ precipitation in an aqueous solution from aluminum salt solutions,^15^ hydrothermal procedures,^2^ solid state decomposition of gibbsite,^16^ sol–gel procedures,^17^ and solvothermal procedures.^2^ Boehmite contains high aggregation of hydroxyl groups on its surface, that supply suable places for modify of its surface with other functional groups such as electrophilic or nucleophilic sites which are enable to immobilization of suitable ligands or metal complexes.^18–22^ Therefore BNPs can be used as an excellent support for fabrication of wide range of heterogeneous catalysts.^2^ BNPs were utilized as support for stabilization of acidic,^23^ basic,^24^ metallic catalysts^25,26^ and organo- or ionic^22^ supported catalysts. More addition, boehmite nanoparticle have several unique attributes such as good surface area, easy availability, non-toxicity, chemical resistance, mechanical strength, thermal stability, good conductivity, high hardness, low cost, excellent biocompatibility, high abrasive and corrosion resistance.^1,2,22^ However, BNPs are also have some disadvantages, such as impurities content (*e.g.* nitrate ions) that led to lower their crystallinity. This impurities concentration may affect properties of the surface property and pore structure of boehmite. In the other hand, BNPs may converts into a γ-Al_2_O_3_ in the high temperatures, but this cannot effect on the catalysis application of BNPs in organic reactions. Because organic reactions take place at temperatures lower than the BNPs phase change. Therefore, Boehmite nanomaterials have also attracted attention in absorbent,^27^ coatings,^28^ flame retardant,^29^ optical material,^30^ ceramics,^31^ vaccine adjuvants,^32^ cosmetic products,^2,33^ pillared clays and sweep-flocculation for fresh water treatment.^13^ Consequently, we investigated a new complex of cobalt with 1,3-bis(pyridin-3-ylmethyl)thiourea on boehmite nanoparticle (Co-(PYT)_2_@BNPs) as a reusable nanocatalyst in the synthesis of tetrazole derivatives. Because tetrazole compounds are an important group of medicinal and organic compounds which possess many uses in several fields such as coordination chemistry, synthetic organic chemistry, drugs, medicinal chemistry as surrogates for carboxylic acids, the photographic industry, catalysis technology, and organometallic chemistry as effective stabilizers of metallopeptide structures.^34–41^

## 2 Experimental

### 2.1 Materials and instruments

Solvents and chemical materials in this project bought from Iranian companies, Aldrich, Merck or Fluka and used sans any purification.

The particle morphology and particle diameters of synthesized catalyst studied *via* FESEM-TESCAN MIRA III Scanning-Electron-Microscope (SEM) from Czechia. In addition, FESEM-TESCAN MIRA III used for type, content and number of elements (*via* WDX and SEM-EDS analysis) of the nanocatalyst. XRD diffraction of the nanocatalyst recorded by a PW1730 device madding Philips Company of Netherlands. IR spectra recorded using KBr pills in a VRTEX 70 model Bruker IR spectrometer. TGA diagram of the nanocatalyst recorded by a SDT Q600 V20.9 Build 20 Thermal Analysis device under air atmosphere in the temperature range of 30–800 °C. NMR spectra of the tetrazoles registered *via* Bruker-DRX-400 spectrometer.

### 2.2 Synthesis of 1,3-bis(pyridin-3-ylmethyl)thiourea ((PYT)_2_) ligand (3)

**Scheme 1:**
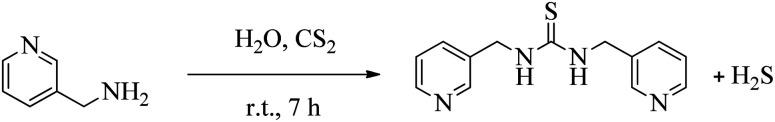
Synthesis of (PYT)_2_ ligand (3).

In a round-bottomed flask, 3-(aminomethyl)pyridine (1, 10 mmol) added to CS_2_ (5 mmol) in H_2_O and stirred at room temperature for 7 h (Scheme 1). The reaction progress consecutively checked by TLC (EtOAc: *n*-hexane, 1 : 2). Since this reaction is exothermic, the temperature increases during the reaction and so this temperature is sufficient for release H_2_S (confirmed by smell and blackening of lead acetate paper). After performance of the reaction, the water-insoluble product filtered, and then recrystallized from hot water and ethanol (1 : 1 v/v).

The structure of (PYT)_2_ ligand was characterized by ^1^H NMR and FT-IR spectroscopies:

#### 2.2.1 1,3-bis(pyridin-3-ylmethyl)thiourea ((PYT)_2_)

^1^H NMR (400 MHz, DMSO-*d*_6_): *δ*_H_ = 5.50 (s, 2H), 8.46–8.44 (d, *J* = 8 Hz, 2H), 8.22 (br, 2H), 7.69–7.66 (d, *J* = 12 Hz, 2H), 7.37–7.33 (d of d, *J* = 8 Hz, *J* = 4 Hz, 2H), 4.69 (s, 4H) ppm.

IR (KBr) cm^−1^: 3272, 3184, 3000, 2923, 2853, 2359, 1913, 1529, 1473, 1422, 1298, 1237, 1193, 1101, 1027, 973, 918, 805, 770, 708, 616, 535.

### 2.3 Synthesis of the catalyst

**Scheme 2:**
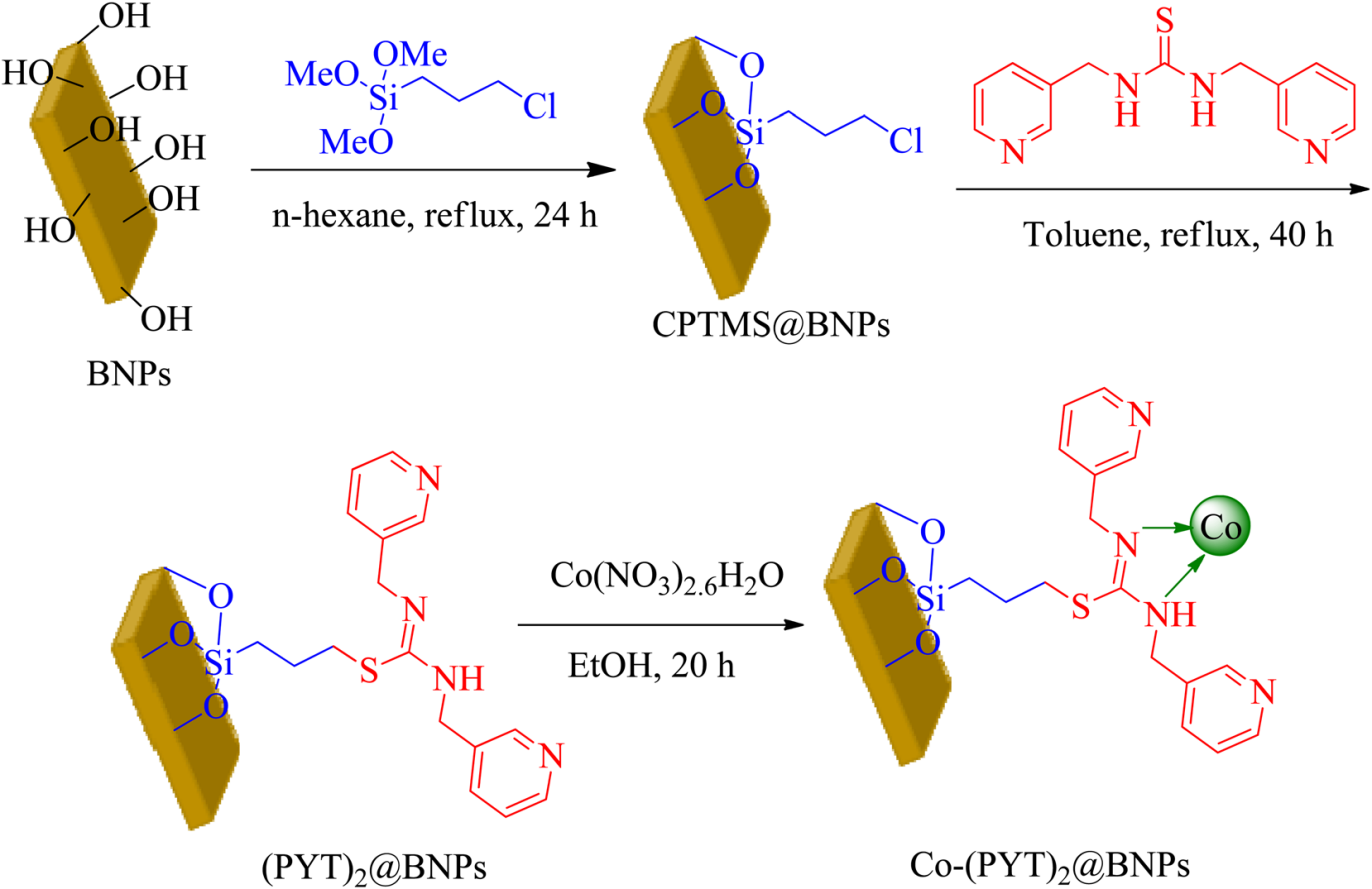
Synthesis of Co-(PYT)_2_@BNPs.

50 mL of aqueous solution of sodium hydroxide (6.490 g) was added to 30 mL of aqueous solution of aluminum nitrate (20 g) as drop to drop under vigorous stirring. The resulting milky mixture was transferred in the ultrasonic bath (for 3 h at room temperature). The resulted BNPs was filtered and washed by distilled water. The obtained BNPs were kept in the oven at 220 °C for 4 h. Then, BNPs were modified by (3-chloropropyl)triethoxysilane (CPTMS) to preparation of CPTMS@BNPs. The CPTMS@BNPs formed matching to reported method in literature.^41,42^ As reported, the BNPs (1.5 g) dispersed in normal hexane, and then CPTMS (2 mL) injected and the mixture stirred for 24 h under reflux conditions that the modified BNPs by CPTMS (CPTMS@BNPs) were produced. The prepared CPTMS@BNPs were filtered, washed by ethanol (EtOH) and dried at room temperature. In order to immobilization of (PYT)_2_ ligand (3) on CPTMS@BNPs, 1 g of CPTMS@BNPs refluxed with (PYT)_2_ in toluene for 40 h. After then, obtained (PYT)_2_@BNPs isolated *via* filtration, washed by DMSO and EtOH, afterward dried at 60 °C. Finally, (PYT)_2_@BNPs (1 g) was dispersed in EtOH, and then Co(NO_3_)_2_·6H_2_O injected to the obtained mixture and then stirred for 24 h under reflux conditions. The resulting catalyst (Co-(PYT)_2_@BNPs) filtered, washed and dried at 60 °C (Scheme 2).

### 2.4 General procedure for the synthesis of tetrazoles catalyzed by Co-(PYT)_2_@BNPs

**Scheme 3:**
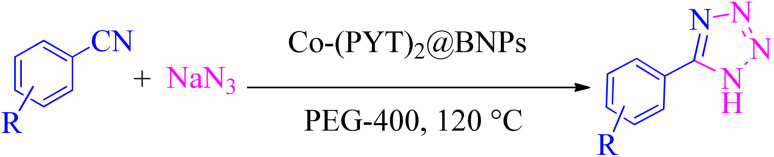
Synthesis of tetrazoles in the attendance of Co-(PYT)_2_@BNPs.

[3 + 2] cycloaddition of NaN_3_ with organic nitrile derivatives was used for the formation of tetrazoles in the attendance of Co-(PYT)_2_@BNPs as nanocatalyst. In this regard, NaN_3_ (1.4 mmol) and nitrile (1 mmol) stirred in the attendance of Co-(PYT)_2_@BNPs (50 mg) in PEG-400 (2 mL) at 120 °C. In the end of the reaction (which checked by TLC), the mixture cooled and was dilute by H_2_O and ethyl acetate. Co-(PYT)_2_@BNPs nanocatalyst isolated *via* simple filtration. Then, HCl (10 mL, 4 N) added and tetrazoles extracted in ethyl acetate. The ethyl acetate solvent dried by anhydrous sodium sulfate and then evaporated (Scheme 3).

### 2.5 Spectral data

#### 2.5.1 5-Phenyl-1*H*-tetrazole

^1^H NMR (400 MHz, DMSO-*d*_6_): *δ*_H_ = 16.89 (br, 1H), 8.06–8.03 (d of d, *J* = 8 Hz, *J* = 4 Hz, 2H), 7.63–7.58 (m, 3H) ppm.

#### 2.5.2 5-(3-nitrophenyl)-1*H*-tetrazole

^1^H NMR (400 MHz, DMSO-*d*_6_): *δ*_H_ = 17.39 (br, 1H), 8.85–84 (t, *J* = 4 Hz, 1H), 8.50–8.47 (d of t, *J* = 12 Hz, *J* = 4 Hz, 1H), 8.45–8.41 (d of q, *J*(d) = 8 Hz, *J*(q) = 4 Hz, 1H), 7.94–7.89 (t, *J* = 12 Hz, 1H) ppm. ^13^C NMR (400 MHz, DMSO-*d*_6_): *δ*_H_ = 153.9, 147.1, 131.9, 130.0, 125.2, 124.3, 120.3 ppm. IR (KBr) cm^−1^: 3439, 3092, 2923, 2856, 2700, 1734, 1620, 1527, 1464, 1374, 1161, 1070, 991, 864, 816, 728, 665, 449.

#### 2.5.3 2-(1*H*-tetrazol-5-yl)phenol

^1^H NMR (400 MHz, DMSO-*d*_6_): *δ*_H_ = 7.99–7.96 (d of d, *J* = 12 Hz, *J* = 4 Hz, 1H), 7.42–7.37 (t of d, *J* = 12 Hz, 1H), 7.07–7.04 (d, *J* = 12 Hz, 1H), 7.02–6.96 (t, *J* = 12 Hz, 1H) ppm. ^13^C NMR (400 MHz, DMSO-*d*_6_): *δ*_C_ = 155.3, 151.8, 132.5, 128.9, 119.7, 116.3, 110.6 ppm. IR (KBr) cm^−1^:3253, 3058, 2941, 2708, 2565, 1892, 1735, 1610, 1546, 1476, 1393, 1358, 1294, 1230, 1150, 1114, 1067, 808, 742, 681, 538, 465.

## 3 Results and discussion

### 3.1 Characterization of the catalyst

At first step, functionalized BNPs by (3-chloropropyl)trimethoxysilane (CPTMS) was produced based on new reported strategy.^41,42^ Subsequently, a new complex of cobalt was fabricated on the surface of functionalized BNPs (Co-(PYT)_2_@BNPs). The catalytic activity of Co-(PYT)_2_@BNPs was confirmed in the synthesis of tetrazoles. This nanocatalyst was characterized using Scanning Electron Microscope (SEM), X-ray diffraction (XRD), Fourier transform infrared spectroscopy (FT-IR), wavelength dispersive X-ray spectroscopy (WDX), energy dispersive X-ray spectroscopy (EDS), and thermogravimetric analysis (TGA) techniques.

**Fig. 1:**
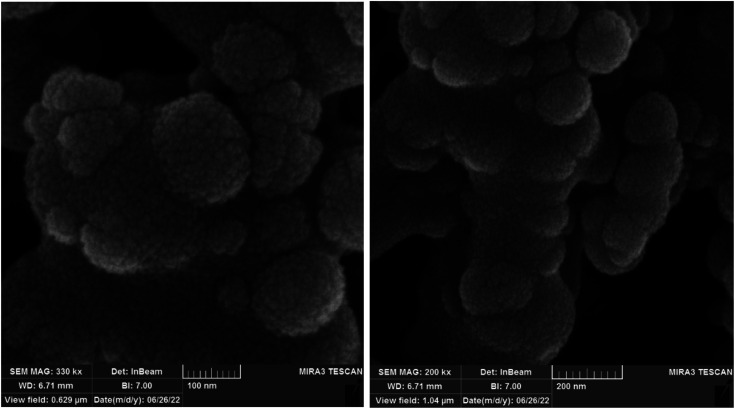
SEM images of Co-(PYT)_2_@BNPs.

The shape, morphology, and diameters size of Co-(PYT)_2_@BNPs studied by FESEM-TESCAN MIRA III Scanning Electron Microscope (SEM) devoice. The SEM images of Co-(PYT)_2_@BNPs illustrated in Fig. 1. As indicate, the particles of Co-(PYT)_2_@BNPs formed in uniform spherical shapes and quite homogeneous diameter less than 70 nm.

**Fig. 2:**
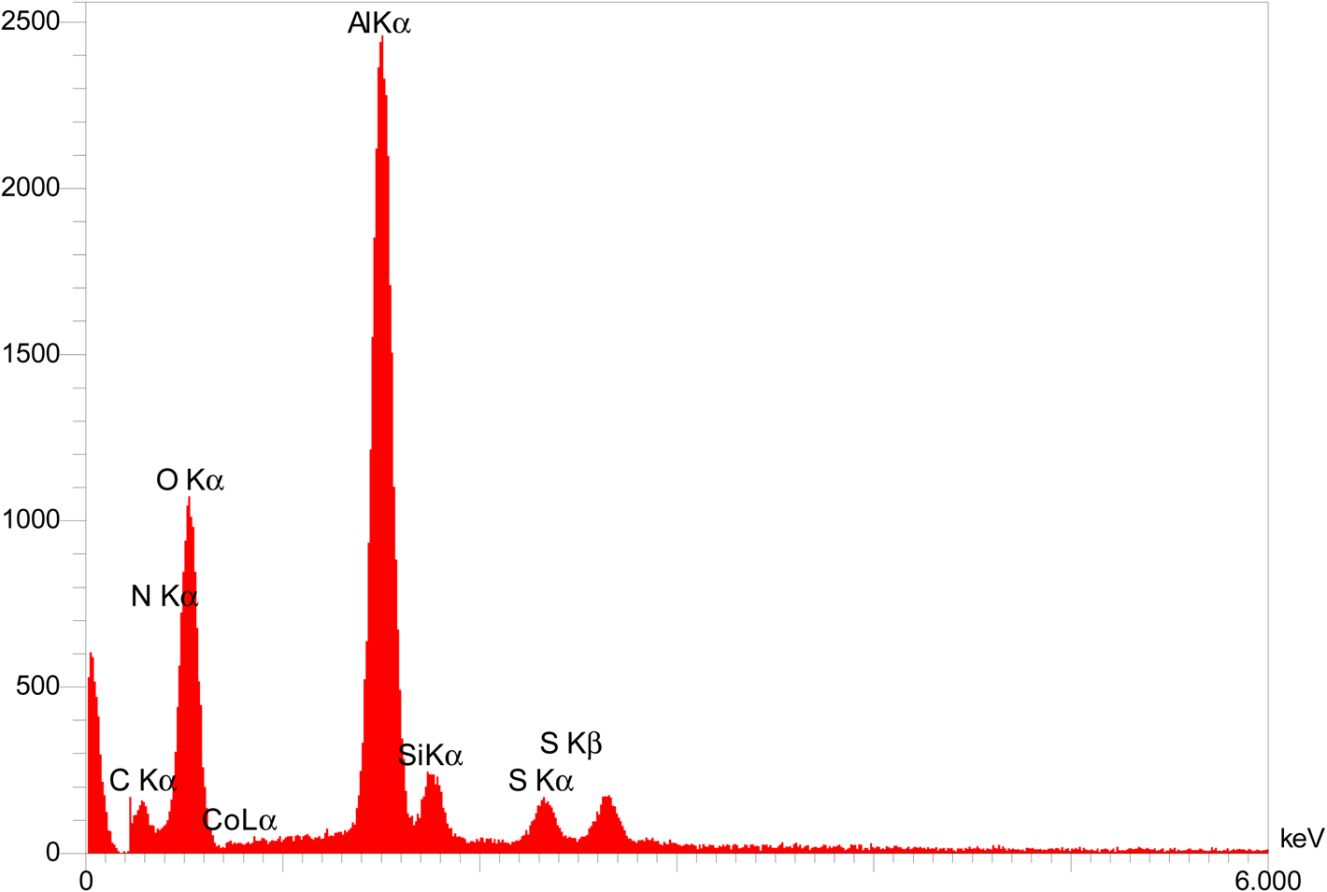
EDS diagram of Co-(PYT)_2_@BNPs.

**Fig. 3:**
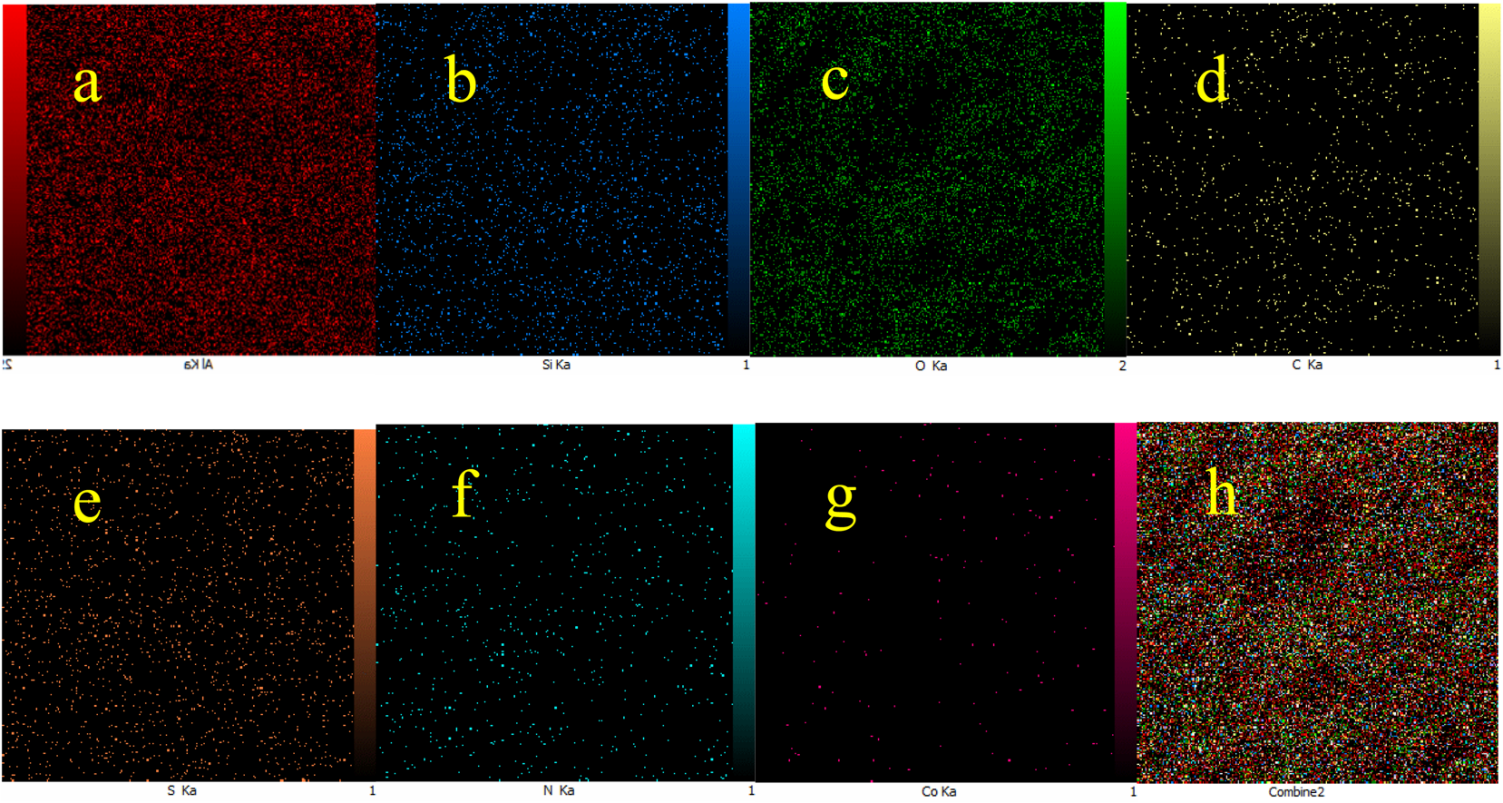
Elemental mapping of (a) aluminum, (b) silicon, (c) oxygen, (d) carbon, (e) sulfur, (f) nitrogen, (g) cobalt and (h) combine of all elements for Co-(PYT)_2_@BNPs.

The obtained results from energy-dispersive X-ray spectroscopy (EDS) analysis of Co-(PYT)_2_@BNPs are summarized in Fig. 2. As shown, Co-(PYT)_2_@BNPs is organize from aluminum, oxygen, silicon, nitrogen, carbon, sulfur and cobalt elements. As accepted, the intensity peaks of Al and O elements is sharped than other elements which are formed skeleton of BNPs. Also, the presence of Si, C, N, S and Co elements indicate the successful stabilization of the cobalt complex on BNPs. Also, wavelength dispersive X-ray spectroscopy (WDX) analysis (Fig. 3) illustrate homogeneous distribution of aluminum, oxygen, silicon, nitrogen, carbon, sulfur and cobalt elements in the structure of Co-(PYT)_2_@BNPs.

**Fig. 4:**
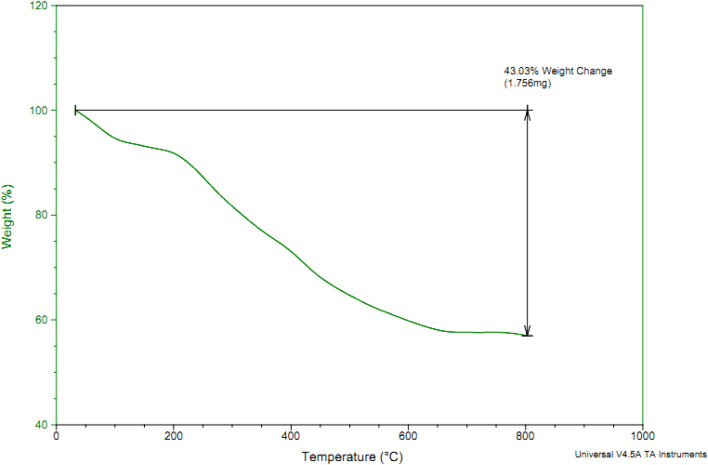
TGA diagram of Co-(PYT)_2_@BNPs.

TGA analysis can used to determine amount of organic and inorganic content in an organic–inorganic composite samples and also can employed to calculate the thermal stability of materials. Therefore, TGA analysis of Co-(PYT)_2_@BNPs was performed from 25 °C to 800 °C within increasing temperature rate of 10 °C min^−1^ under air atmosphere (Fig. 4). In TGA diagram of Co-(PYT)_2_@BNPs, a small weight losses (8% of weight) up to 150 °C is corresponded to the evaporation of solvents.^43^ As shown, any weight loss was not indicate up to 250 °C except evaporation of solvents which showed excellent thermal stability of Co-(PYT)_2_@BNPs. Therefore Co-(PYT)_2_@BNPs can be used as catalyst under hard conditions in wide range of organic reactions. TGA analysis of Co-(PYT)_2_@BNPs illustrated a considerable mass loss (35% of weight) between 250–650 °C which due to the decomposition of immobilized organic layers on the surface of modified BNPs.^44^

**Fig. 5:**
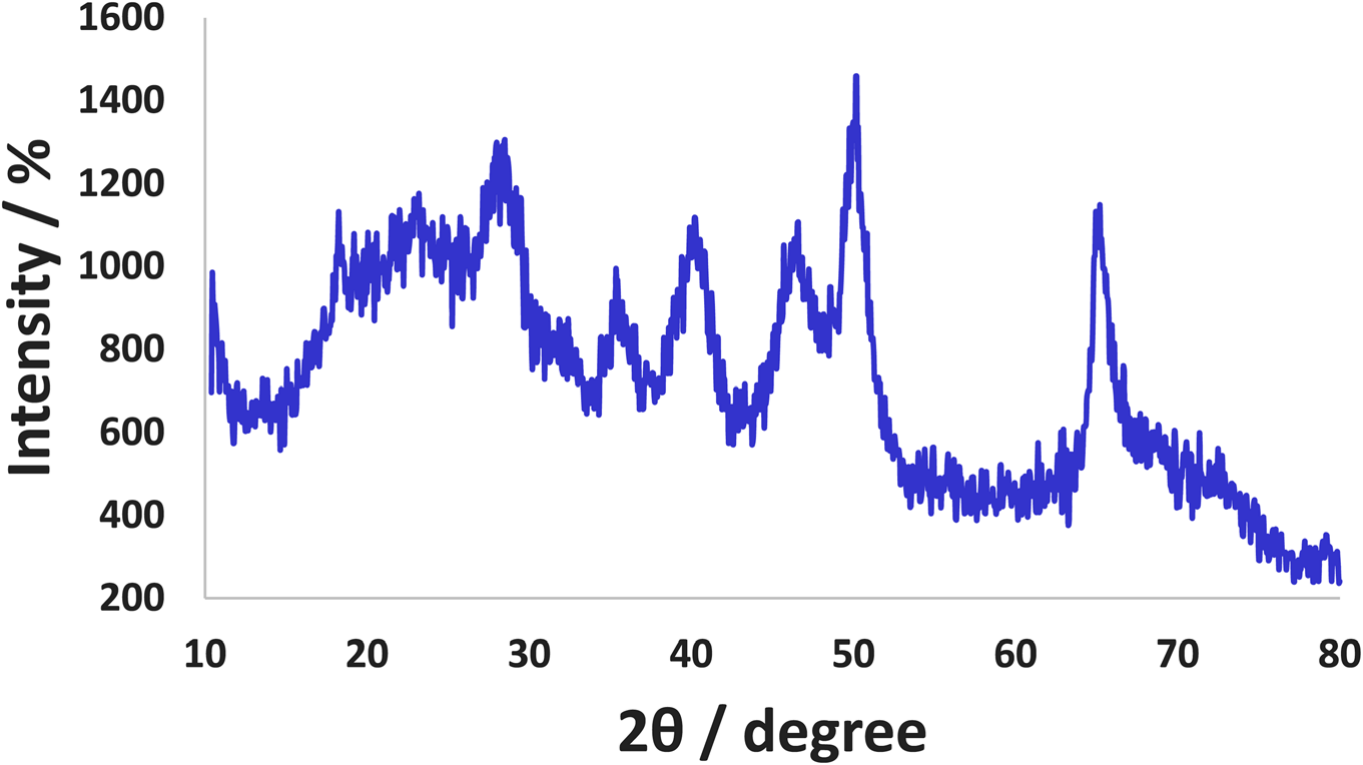
Normal XRD pattern of Co-(PYT)_2_@BNPs.

X-ray diffraction (XRD) pattern of Co-(PYT)_2_@BNPs is obtained with Cu Kα radiation (*λ* = 0.154 nm). As shown in Fig. 5, the XRD pattern of Co-(PYT)_2_@BNPs shows several peaks of 2*θ* = 14.69 (0 2 0), 27.89 (1 2 0), 40.34° (0 3 1), 46.84° (1 3 1), 49.89° (0 5 1), 53.99° (2 0 0), 56.54° (1 5 1), 58.59° (0 8 0), 63.74° (2 3 1), 65.64° (0 0 2), 67.74° (1 7 1), and 72.89° (2 5 1) that confirm BNPs is stable in orthorhombic unit cell^2,4^ after stabilization of cobalt complex. The intensity of all peaks was decreased than BNPs due to the chemical modifications of BNPs.^33^ Also, a broad peak of 2*θ* from 15° to 25° related to the amorphous SiO_2_.^45^ Also, XRD pattern of Co-(PYT)_2_@BNPs showed four peaks at 2*θ* = 15.79° (1 1 0), 32.44° (2 2 0), 54.19° (1 4 1) and 63.14° (5 0 3) which can be related to Cobalt(ii) species.^33^

**Fig. 6:**
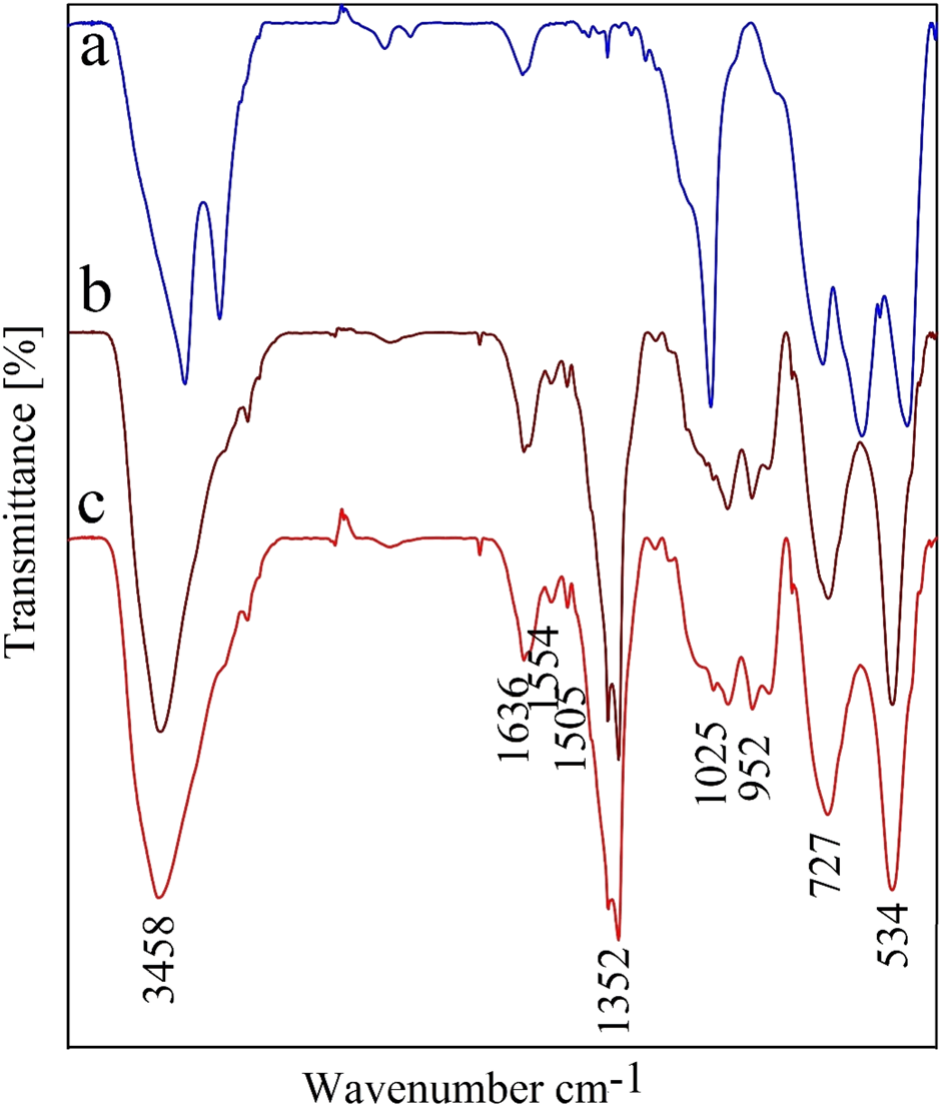
FT-IR spectra of (a) CPTMS@BNPs, (b) (PYT)_2_@BNPs, and (c) Co-(PYT)_2_@BNPs.

The FT-IR spectrum of CPTMS@BNPs, (b) (PYT)_2_@BNPs, and (c) Co-(PYT)_2_@BNPs shown in Fig. 6. Bands vibration at low wavenumbers <750 cm^−1^ in the FT-IR spectra related to the vibrations of the Al–O bonds.^4^ O–H and N–H bands appeared above 3000 cm^−1^ in the FT-IR spectra.^46^ In addition, the stretching vibrations of Si–O identified in region 805 cm^−1^ and 1075 cm^−1^ of FT-IR spectra.^41,47^ In addition, stretching vibrations of the C=N groups have appeared in the 1635 cm^−1^ region.^4,48^

### 3.2 Catalytic studying of the catalyst

After characterization of Co-(PYT)_2_@BNPs, it was used as efficient, recyclable and biocompatible nanocatalyst in the synthesis of tetrazole heterocyclic compounds. The best reaction conditions obtained through [3 + 2] cycloaddition of NaN_3_ and benzonitrile as model reaction (Table 1). The model reaction did not taken place in the absent of Co-(PYT)_2_@BNPs nanocatalyst (Table 1, entry 1). While, the presentence of Co-(PYT)_2_@BNPs is required for the synthesis of 5-substituted 1*H*-tetrazole heterocyclic compounds. As expected, the model reaction occurs with the addition of catalyst and it faster proceeded by increasing in amount of Co-(PYT)_2_@BNPs catalyst. As shown, the model reaction completed within acceptable time when the amount of catalyst increased up to 50 mg (Table 1, entry 3). Among of several solvents (such as H_2_O, DMSO and PEG-400) which are examined, PEG-400 was provided the best results in term of reaction time and isolated yield of the pure product (Table 1, entry 3). Also, the effect of equivalent amount of NaN_3_ to benzonitrile and temperature on the model reaction was studied, which the best results were obtained with 1.4 mmol of NaN_3_ per 1 mmol of benzonitrile at 120 °C (Table 1, entry 3).

**Table 1:** Optimizing the best conditions for the synthesis of tetrazoles in the presence of Co-(PYT)_2_@BNPs nanocatalyst

Entry	Amount of the catalyst (mg)	Solvent	NaN_3_ (mmol)	Time (min)	Temperature (°C)	Yield (%)^a^
1	—	PEG	1.4	150	120	N. R.^b^
2	40	PEG	1.4	310	120	85
3	50	PEG	1.4	100	120	98
4	50	PEG	1.3	120	120	80
5	50	DMSO	1.4	100	120	81
6	50	H_2_O	1.4	100	Reflux	20
7	50	PEG	1.4	100	100	49

a Isolated yield within 120 min.

b No reaction.

The scope of catalytic application of Co-(PYT)_2_@BNPs nanocatalyst was extended in the [3 + 2] cycloaddition of NaN_3_ and other benzonitrile derivatives (Table 2). In this regard, several benzonitrile compounds with an electron-withdrawing or electron-donating groups on *para*- *meta*- or *ortho*-position of aromatic ring were examined under optimized reaction conditions in hand. As shown in Table 2, all corresponding heterocyclic tetrazoles were produced in good yields. Also, phthalonitrile was employed as nitrile substrate which has two similar cyano groups on 1,2 position of its aromatic ring (Table 2, entry 4). As shown in Table 2 (entry 4), this methodology was provided only monoaddition which may be related to steric hindrance or selectivity of this catalyst. Also [1,1′-biphenyl]-4-carbonitrile (4-phenyl benzonitrile) was synthesized based on recently reported literature^49^ and it was investigated in the [3 + 2] cycloaddition reaction with NaN_3_ (Table 2, entry 11).

**Table 2:** Synthesis of 5-substituted 1*H*-tetrazole derivatives catalyzed by Co-(PYT)_2_@BNPs nanocatalyst

Entry	Nitrile	Product	Time (min)	Yield (%)^a^	Melting point	Reference
1	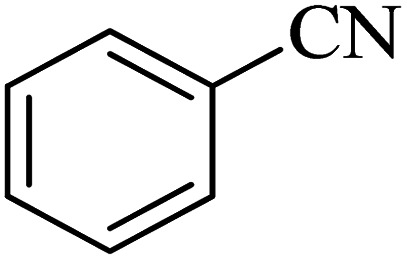	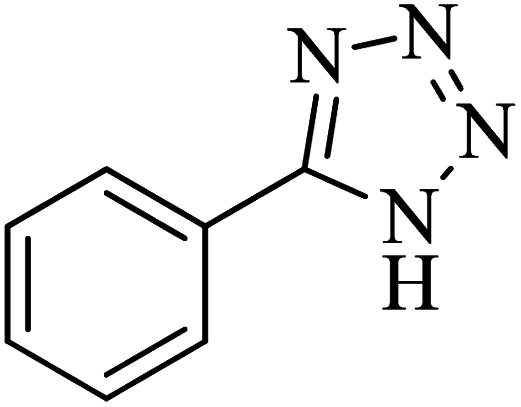	120	98	214–215	36
2	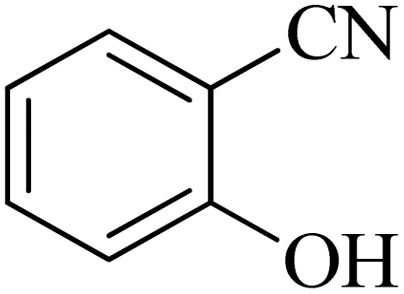	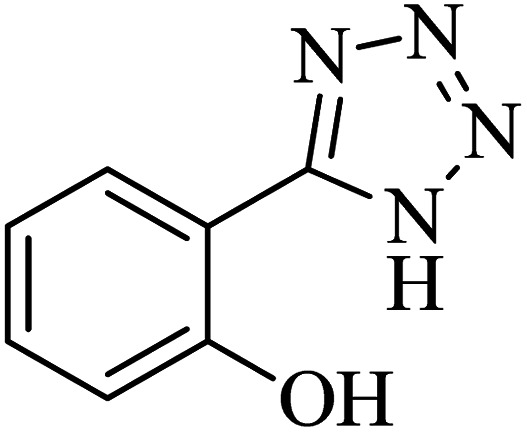	180	94	223–226	41
3	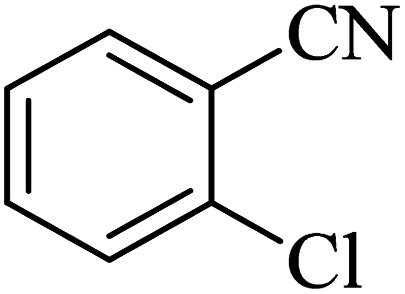	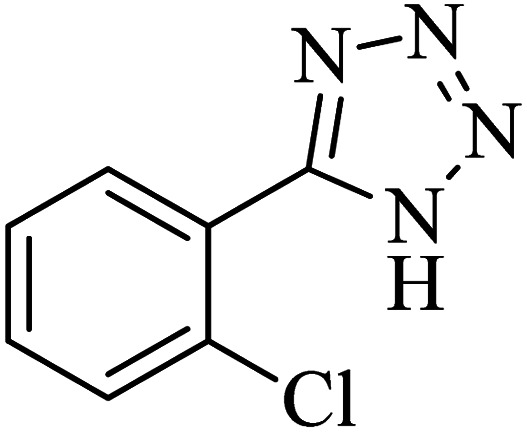	200	95	179–181	36
4	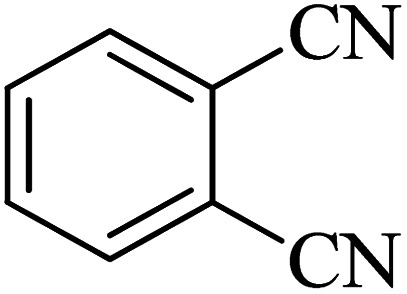	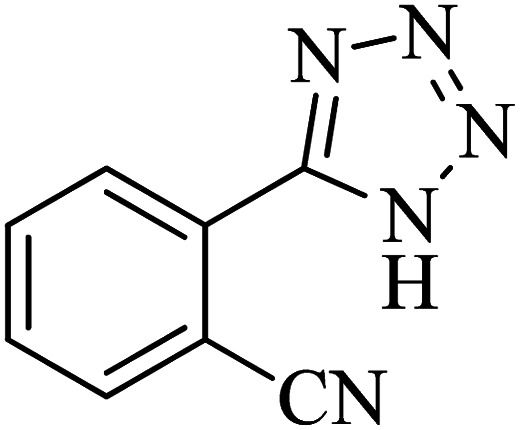	50	93	210–211	41
5	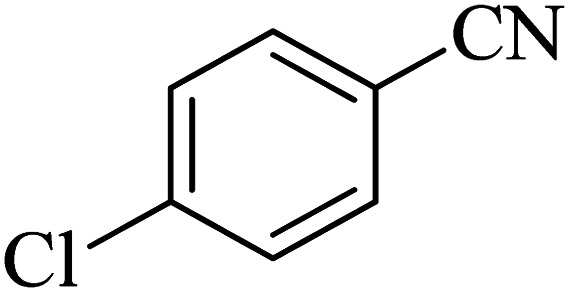	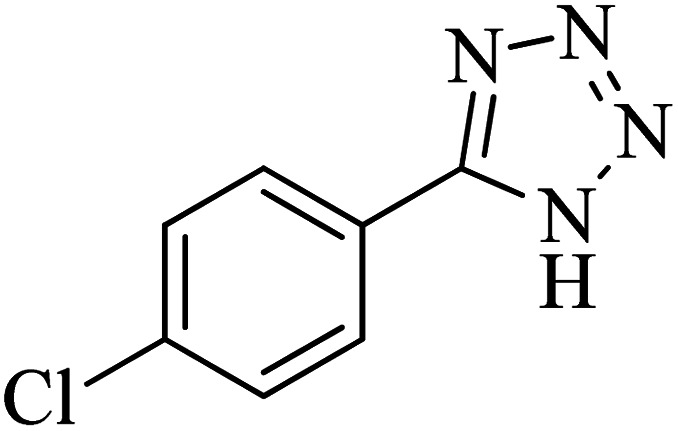	190	96	261–262	36
6	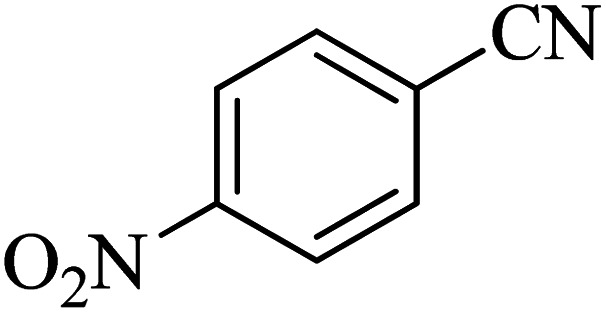	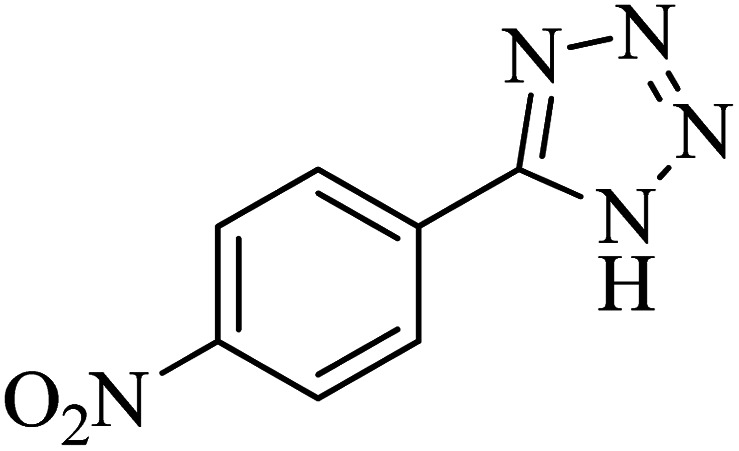	405	98	217–220	40
7	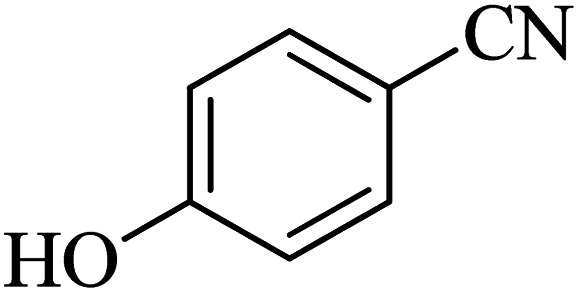	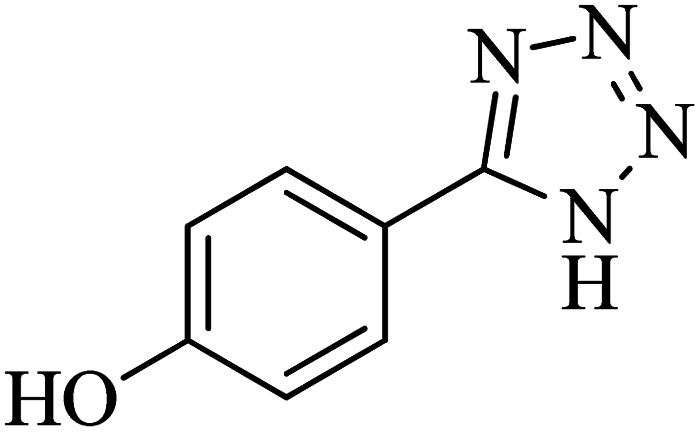	50	93	229–231	40
9	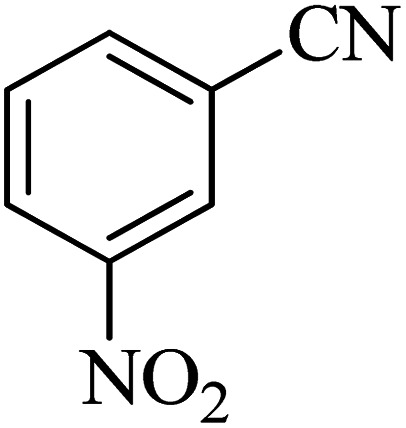	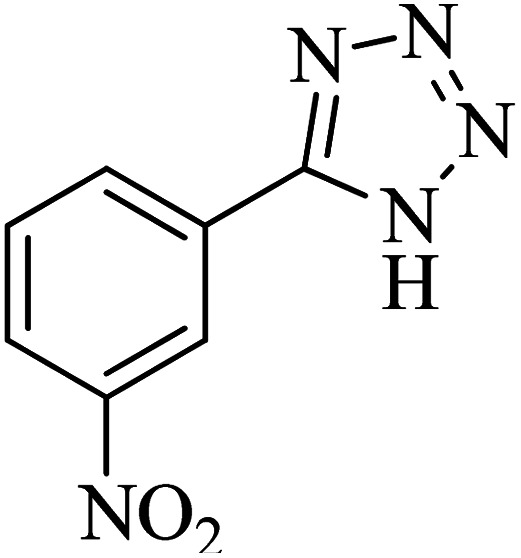	360	95	149–151	44
10	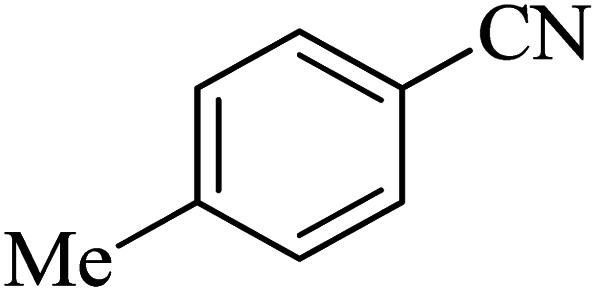	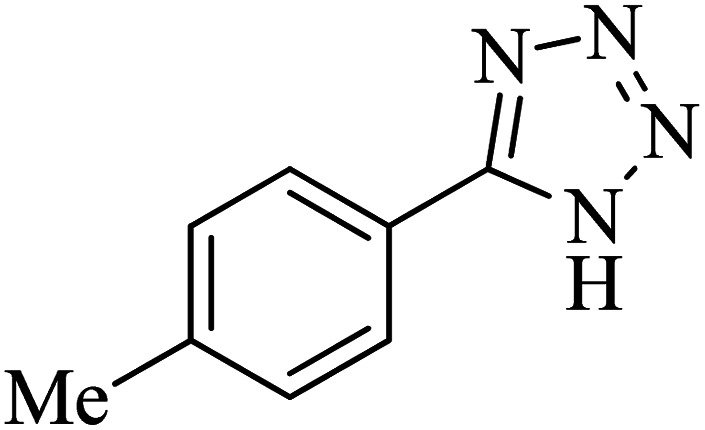	16 h	89	247–249	50 and 51
11	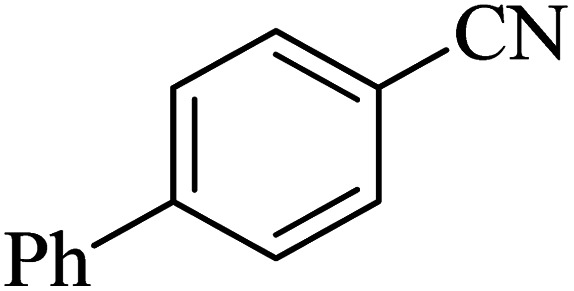	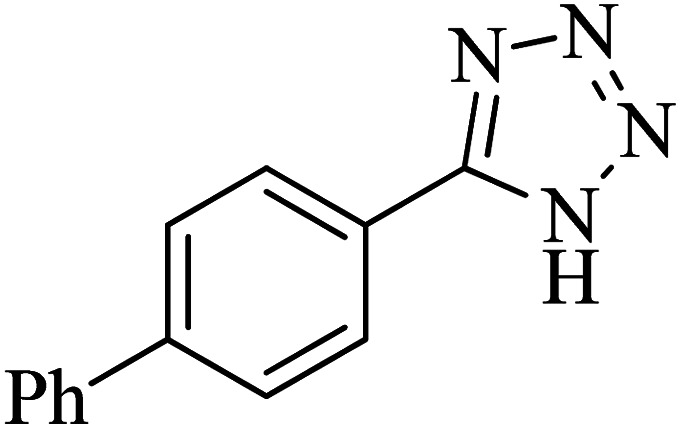	46 h	71	245–248	52 and 53

a Isolated yield.

**Scheme 4:**
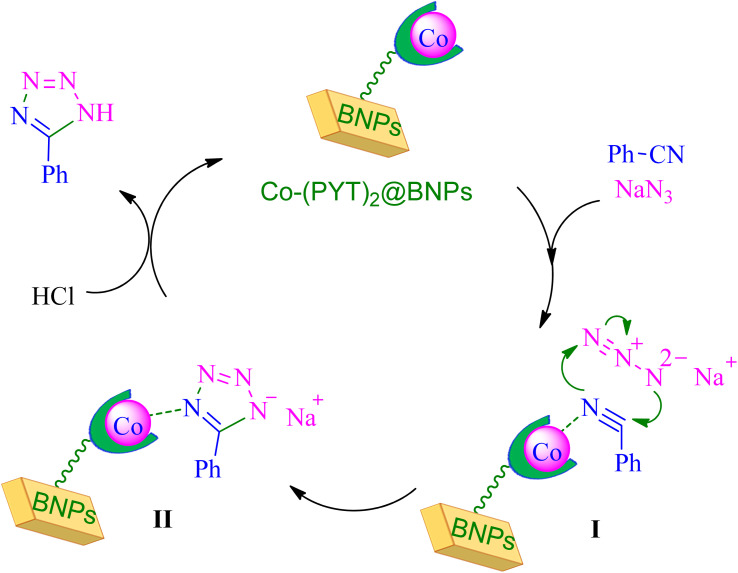
Expected mechanism for the synthesis of tetrazoles in the presence of Co-(PYT)_2_@BNPs nanocatalyst.

Based on reported authentic methodologies about synthesis of tetrazoles in the presence of immobilized transition metal catalysts,^46,54^ a mechanism cycle for the synthesis of tetrazoles in the presence of Co-(PYT)_2_@BNPs catalyst offered in Scheme 4.

### 3.3 Reusability of the catalyst

**Fig. 7:**
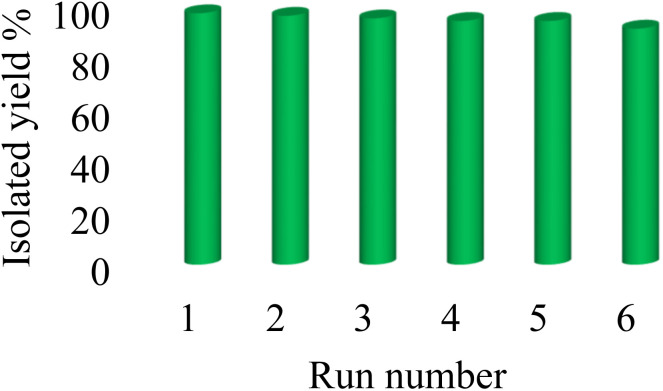
The reusability of Co-(PYT)_2_@BNPs in the synthesis of 5-phenyl-1*H*-tetrazole.

As mentioned, Co-(PYT)_2_@BNPs catalyst is stable and it has heterogeneity nature. Therefore the reusability and retrievability of Co-(PYT)_2_@BNPs nanocatalyst were investigated in the [3 + 2] cycloaddition of benzonitrile and NaN_3_ for the synthesis of 5-phenyl-1*H*-tetrazole. As shown in Fig. 7, Co-(PYT)_2_@BNPs catalyst can be recovered and reused up to 6 runs without any further activation.

### 3.4 Comparison of the catalyst

The efficiency and advantages of Co-(PYT)_2_@BNPs catalyst than previous reported catalysts were compared in the [3 + 2] cycloaddition of benzonitrile with sodium azide in the presence of Co-(PYT)_2_@BNPs and previous catalysts (Table 3). As shown, Co-(PYT)_2_@BNPs catalyst afford 98% of 5-phenyl-1*H*-tetrazole product in 2 h which is better than previous reported catalysts in terms of time and yields. Also, some of previous catalysts have several disadvantages, limitations or drawbacks such as low yield of the products, long reaction times, expensive catalysts, non-environmental conditions, non or difficult separation of the catalysts and utilize hazard solvents. While, in this work, the synthesis of tetrazoles was introduced in the presence of Co-(PYT)_2_@BNPs as reusable catalyst in green solvent such as PEG, in short reaction time with acceptable yield.

**Table 3:** Comparison results of Co-(PYT)_2_@BNPs nanocatalyst with other catalysts for synthesis of 5-phenyl-1*H*-tetrazole

Entry	Catalyst	Time (h)	Yield (%)	Ref.
1	CoY zeolite	14	90	37
2	Cu–Zn alloy nanopowder	10	95	55
3	B(C_6_F_5_)_3_	8	94	56
4	Fe_3_O_4_@SiO_2_/Salen Cu(ii)	7	90	57
5	Fe_3_O_4_/ZnS HNSs	24	81.1	58
6	Pd-isatin-boehmite	8	94	59
7	Mesoporous ZnS	36	86	60
8	AgNO_3_	5	83	61
9	CuFe_2_O_4_	12	82	62
10	Nano ZnO/Co_3_O_4_	12	90	63
11	Pd-SMTU@boehmite	2.5	95	64
12	Cu-TBA@biochar	7	98	41
13	l-cysteine-Pd@MCM-41	3	98	65
14	Ni-MP(AMP)_2_@Fe-biochar	3.8	92	34
15	Cu(ii)-adenine-MCM-41	5	92	66
16	Pd-Arg@boehmite	7	97	36
17	Cu-DABP@Fe_3_O_4_/MCM-41	2	99	46
18	Fe_3_O_4_@boehmite NPs	4	97	67
19	Co-(PYT)_2_@BNPs	2	98	This work

## 4 Conclusions

In Conclusion, we synthesized a new stabilized complex of cobalt on modified boehmite NPs by 1,3-bis(pyridin-3-ylmethyl)thiourea (Co-(PYT)_2_@BNPs) as highly practical, retrievable, stable, and maintainable organic–inorganic hybrid nanocatalyst. Co-(PYT)_2_@BNPs was characterized by various techniques such as XRD, TGA, SEM, EDS, WDX and FT-IR. Catalytic activity of this catalyst was studied in the formation of organic heterocyclic compounds such as tetrazole derivatives. Co-(PYT)_2_@BNPs display high activity, stability and recyclability in the synthesis of tetrazoles.

## Conflicts of interest

There are no conflicts to declare.

## Supplementary Material

RA-013-D2RA07510E-s001
