# Supplementary material for: Synthesis of tetrazoles catalyzed by a new and recoverable nanocatalyst of cobalt on modified boehmite NPs with 1,3-bis(pyridin-3-ylmethyl)thiourea
Source: RSC Adv. 2023 Mar 17;13(13):8890–900. doi: 10.1039/d2ra07510e (PMC10020908; doi:10.1039/d2ra07510e)

Electronic Supplementary Material (ESI) for RSC Advances.
This journal is © The Royal Society of Chemistry 2023

Supplementary Data

Synthesis of tetrazoles catalyzed by a new and recoverable nanocatalyst of cobalt on modified boehmite NPs with 1,3-bis(pyridin-3-ylmethyl)thiourea

Arida Jabbari[*,a], Parisa Moradi[b], Bahman Tahmasbi[b]

[a]*Department of Chemistry, Qeshm Branch, Islamic Azad University, Qeshm, Iran.*

[b]*Department of Chemistry, Faculty of Science, Ilam University, P.O. Box 69315516, Ilam, Iran.*

Abstract: At first part of this work, boehmite nanoparticles (BNPs) were synthesized from aqueous solutions of NaOH and $Al(NO_3)_3.9H_2O$. Then, BNPs surface was modified using 3-choloropropyltrimtoxysilane (CPTMS) and then 1,3-bis(pyridin-3-ylmethyl)thiourea ($(PYT)_2$) was anchored on the surface of modified BNPs (CPTMS@BNPs). In the final step, a complex of cobalt stabilized on its surface (Co-$(PYT)_2$@BNPs). The final obtained nanoparticles were characterized by FT-IR spectra, TGA analysis, SEM imaging, WDX analysis, EDS analysis, and XRD pattern. At second part, Co-$(PYT)_2$@BNPs used as a highly efficient, retrievable, stable, and organic-inorganic hybrid nanocatalyst for homoselective formation of organic heterocyclic compounds such as tetrazole derivatives. The homoselectivity of Co-$(PYT)_2$@BNPs was confirmed in the [3+2]cycloaddition of phthalonitrile and sodium azide (NaN_3). Co-$(PYT)_2$@BNPs as a novel nanocatalyst is stable and it has heterogeneity nature nanocatalyst; therefore, it can recovered and reused again for several consecutively runs without any re-activation.

Keywords: Boehmite nanoparticles; Heterogeneous catalyst; Homoselective nanocatalyst, Heterocyclic tetrazoles, Cobalt complex.

* Address correspondence to A. Jabbari, Department of Chemistry, Qeshm Branch, Islamic Azad University, Qeshm, Iran. E-mail address: arida_jabbari@yahoo.com

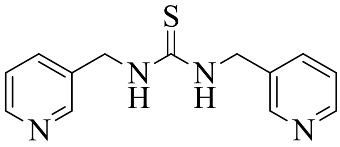

3

1,3-bis(pyridin-3-ylmethyl)thiourea ((PYT)$_2$): ^{1}H NMR (400 MHz, DMSO-d6): δ_H= 5.50 (s, 2H), 8.46-8.44 (d, *J*= 8 Hz, 2H), 8.22 (br, 2H), 7.69-7.66 (d, *J*= 12 Hz, 2H), 7.37-7.33 (d of d, *J*= 8 Hz, *J*= 4 Hz, 2H), 4.69 (s, 4H) ppm.

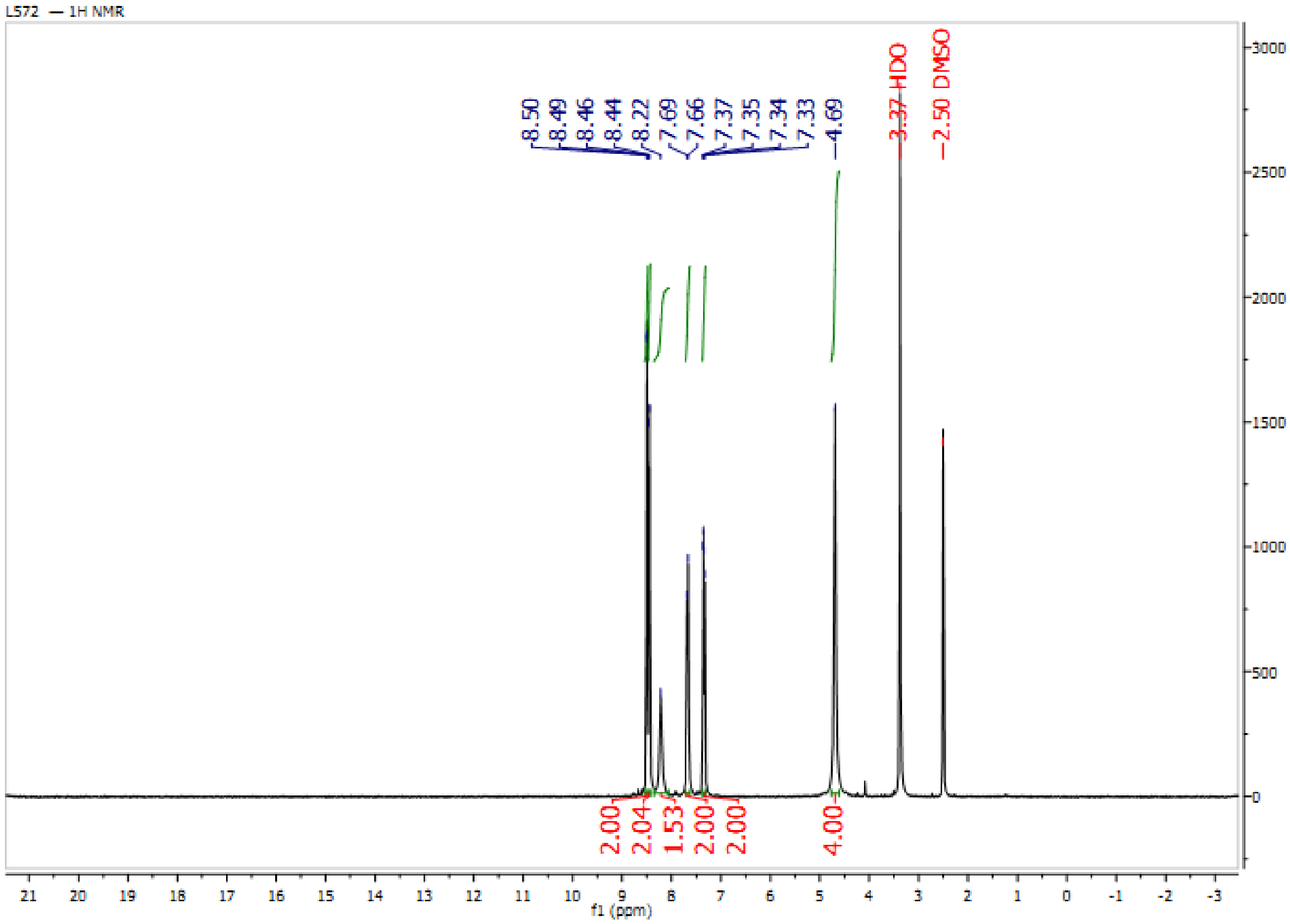

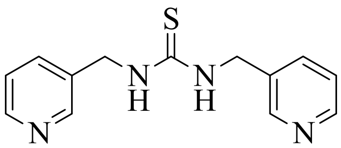

3

1,3-bis(pyridin-3-ylmethyl)thiourea ((PYT)$_2$): ^{1}H NMR (400 MHz, DMSO-d6): δ_H= 5.50 (s, 2H), 8.46-8.44 (d, *J*= 8 Hz, 2H), 8.22 (br, 2H), 7.69-7.66 (d, *J*= 12 Hz, 2H), 7.37-7.33 (d of d, *J*= 8 Hz, *J*= 4 Hz, 2H), 4.69 (s, 4H) ppm.

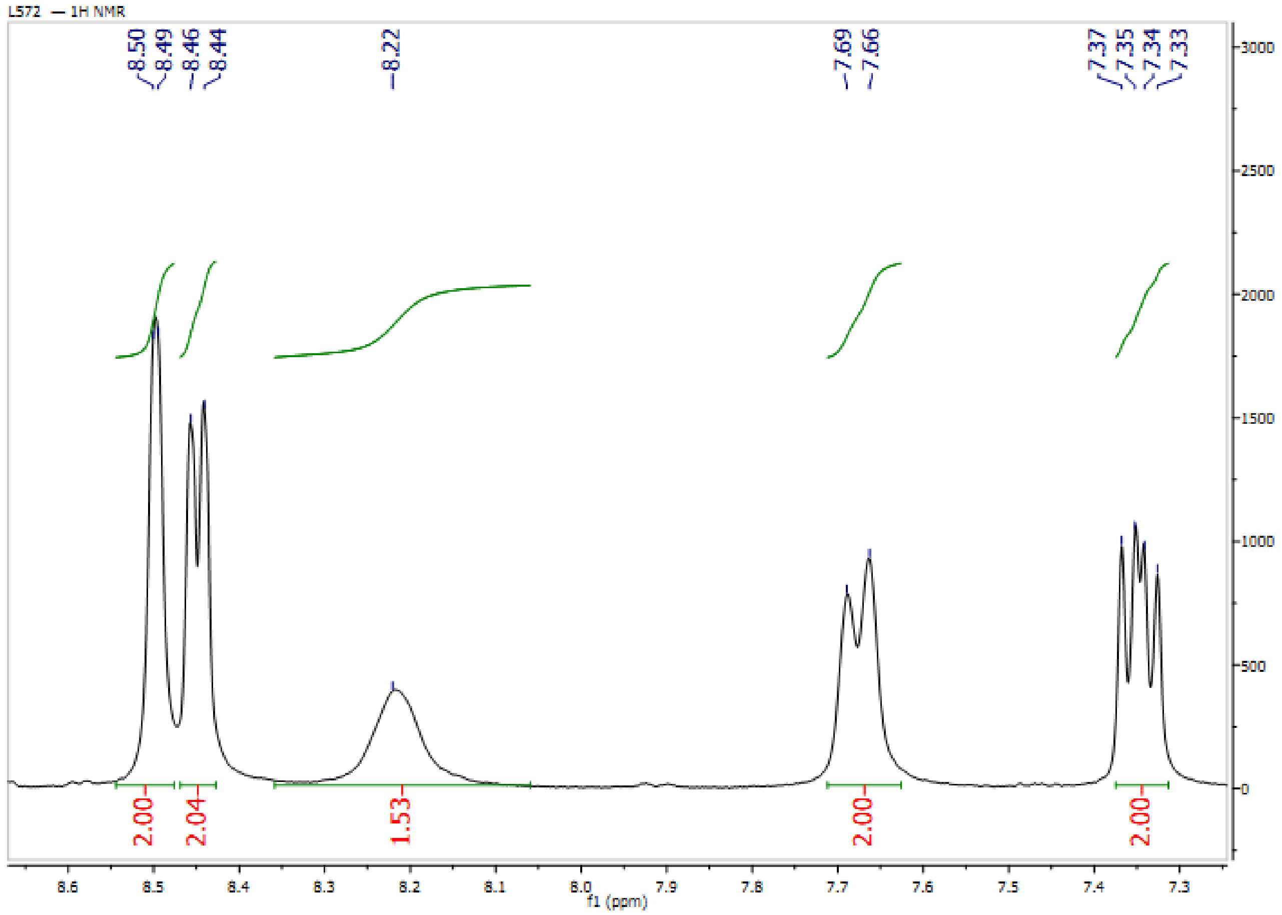

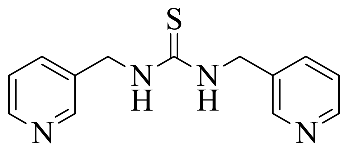

3

1,3-bis(pyridin-3-ylmethyl)thiourea ((PYT)$_2$): IR (KBr) cm^{-1}: 3272, 3184, 3000, 2923, 2853, 2359, 1913, 1529, 1473, 1422, 1298, 1237, 1193, 1101, ,1027, 973, 918, 805, 770, 708, 616, 535.

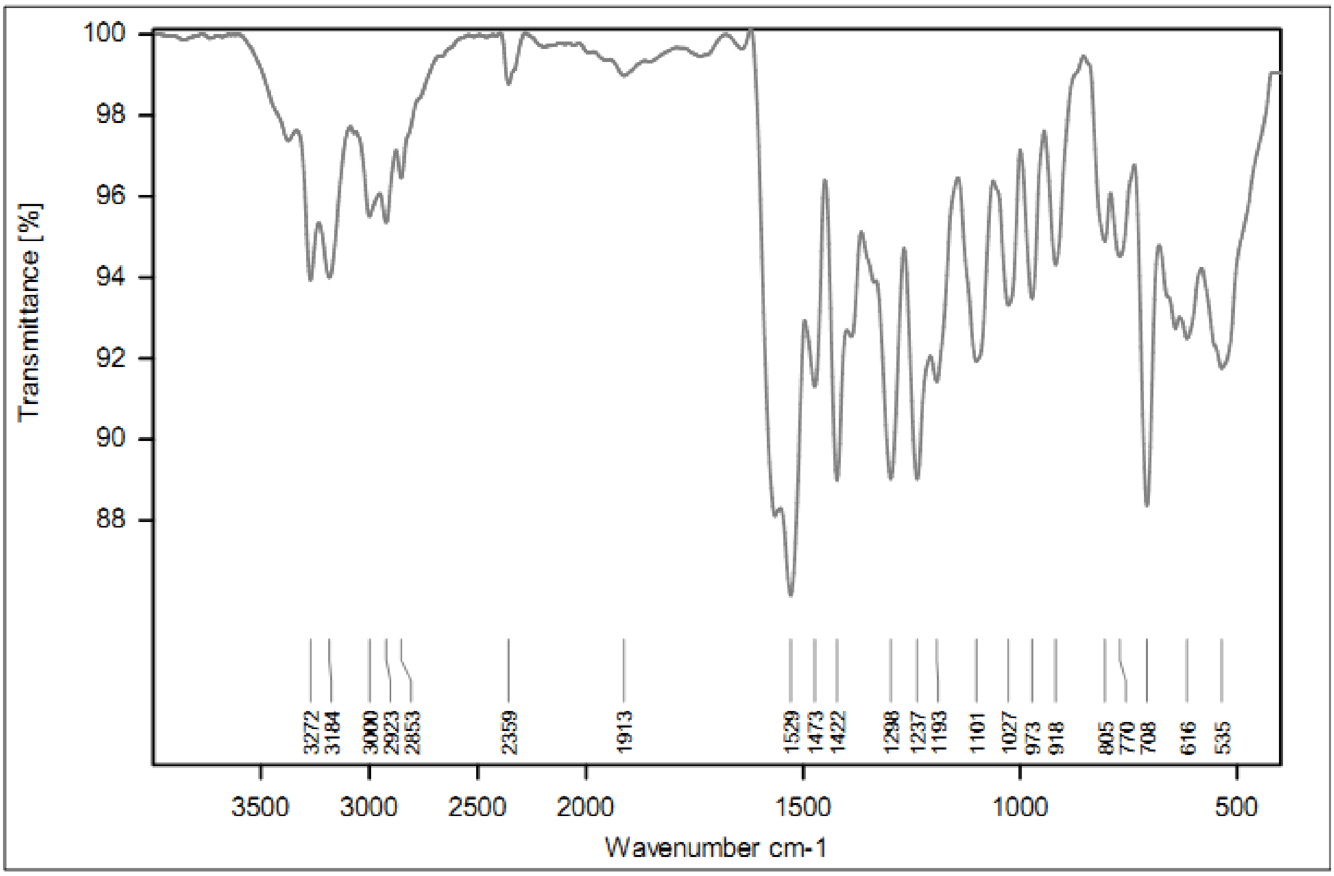

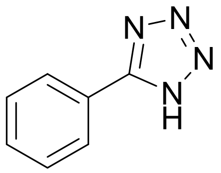

5-phenyl-1H-tetrazole: ^{1}H NMR (400 MHz, DMSO-d6): δ_H= 16.89 (br, 1H), 8.06-8.03 (d of d, *J*= 8 Hz, *J*= 4 Hz, 2H), 7.63-7.58 (m, 3H) ppm.

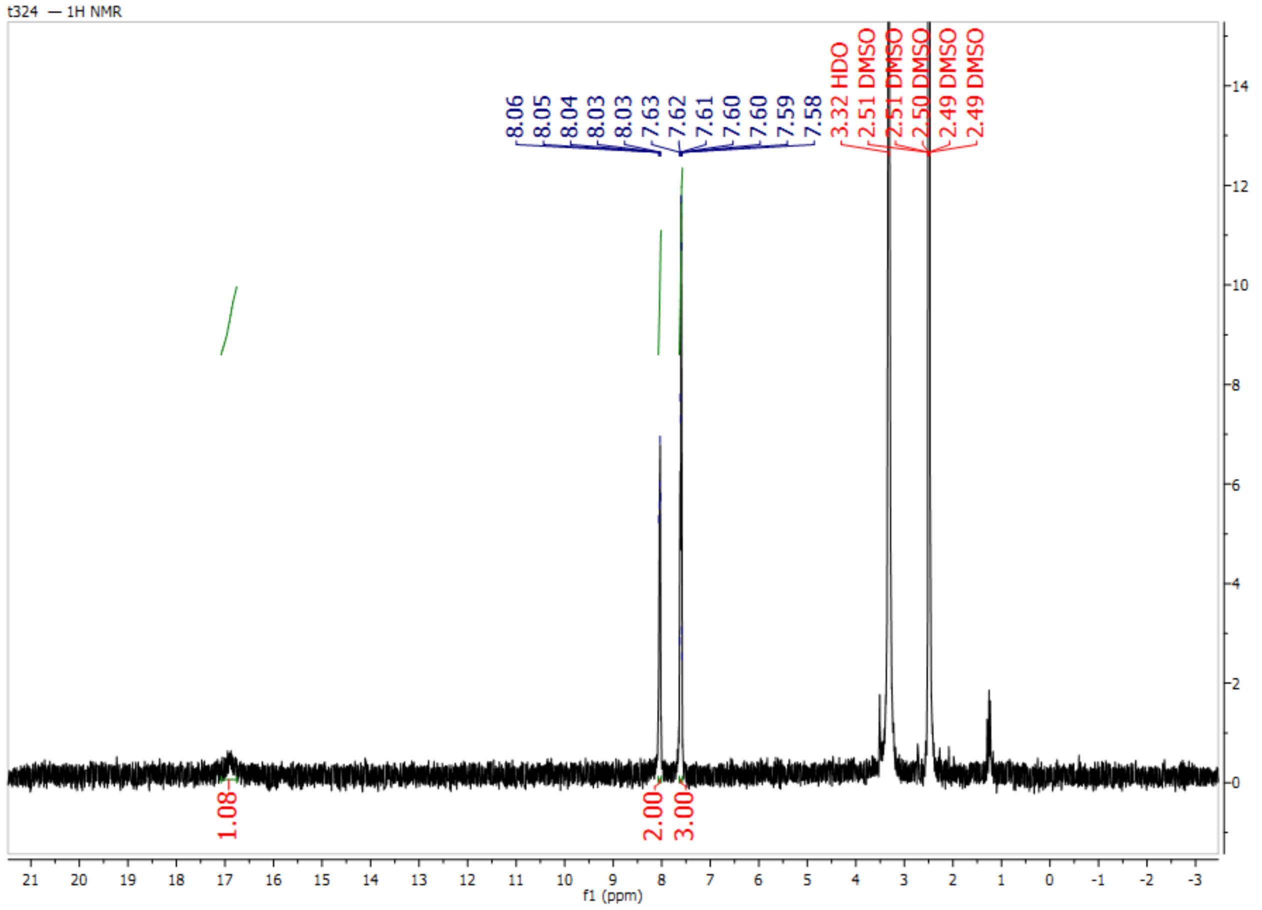

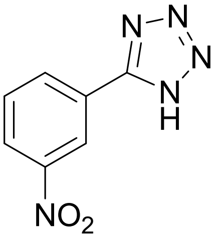

5-(3-nitrophenyl)-1H-tetrazole: ^{1}H NMR (400 MHz, DMSO-d6): δ_H= 17.39 (br, 1H), 8.85-84 (t, *J*= 4 Hz, 1H), 8.50-8.47 (d of t, *J*= 12 Hz, *J*= 4 Hz, 1H), 8.45-8.41 (d of q, *J*(d)= 8 Hz, *J*(q)= 4 Hz, 1H), 7.94-7.89 (t, *J*= 12 Hz, 1H) ppm.

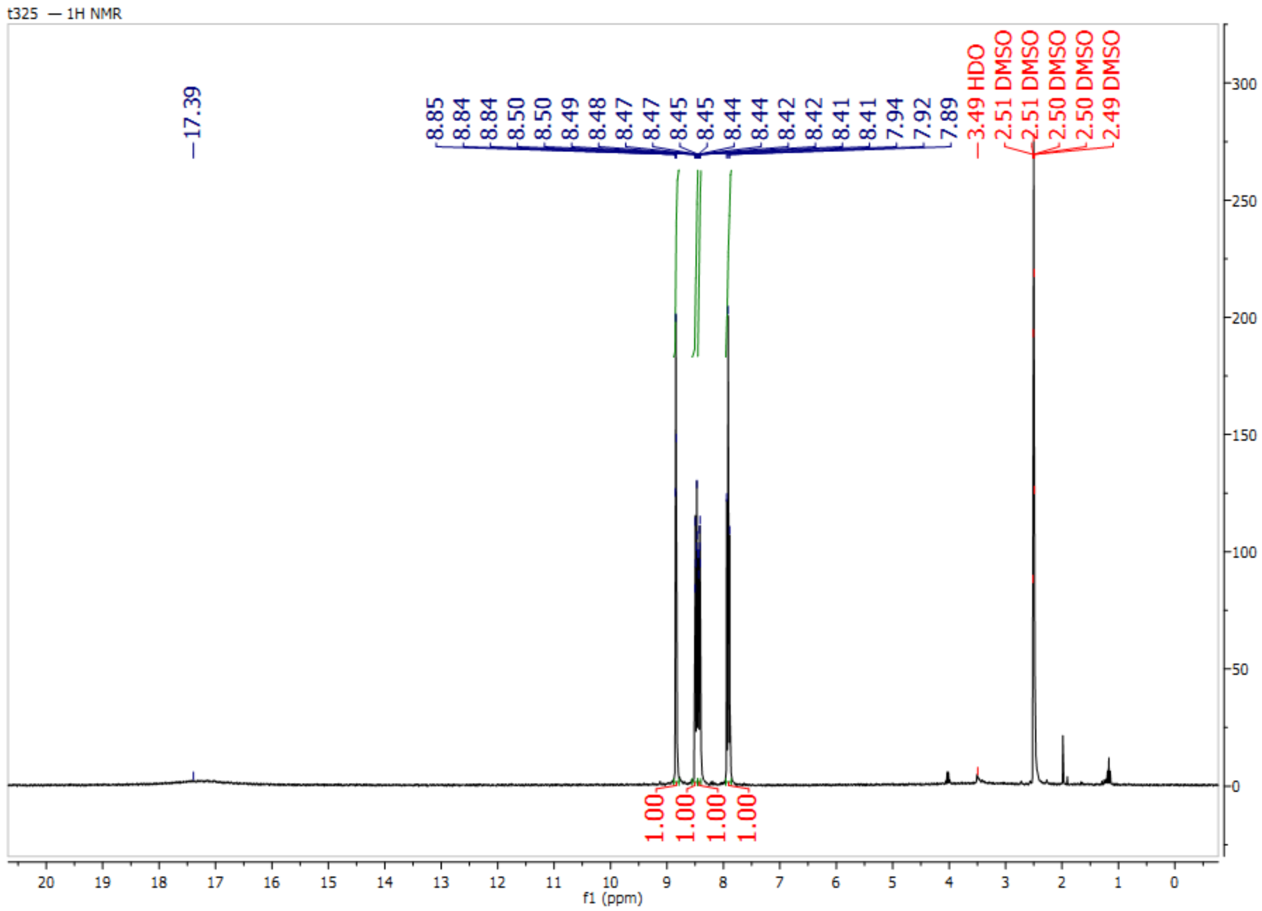

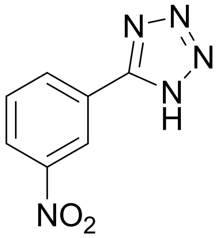

5-(3-nitrophenyl)-1H-tetrazole: ^{13}C NMR (400 MHz, DMSO-d6): δ_C= 153.9, 147.1, 131.9, 130.0, 125.2, 124.3, 120.3 ppm.

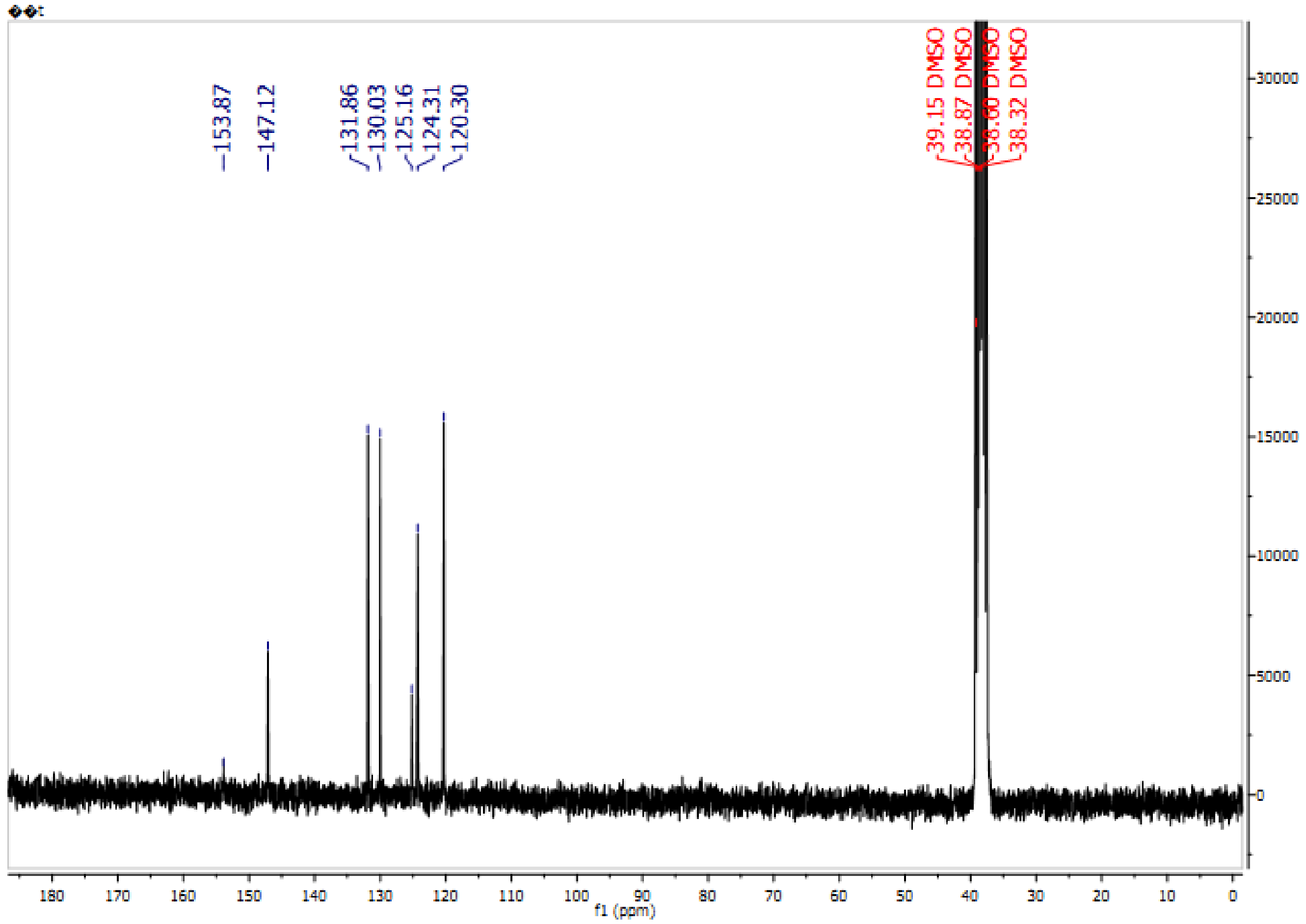

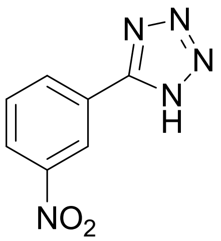

5-(3-nitrophenyl)-1H-tetrazole: IR (KBr) cm^{-1}: 3439, 3092, 2923, 2856, 2700, 1734, 1620, 1527, 1464, 1374, 1161, 1070, 991, 864, 816, 728, 665, 449.

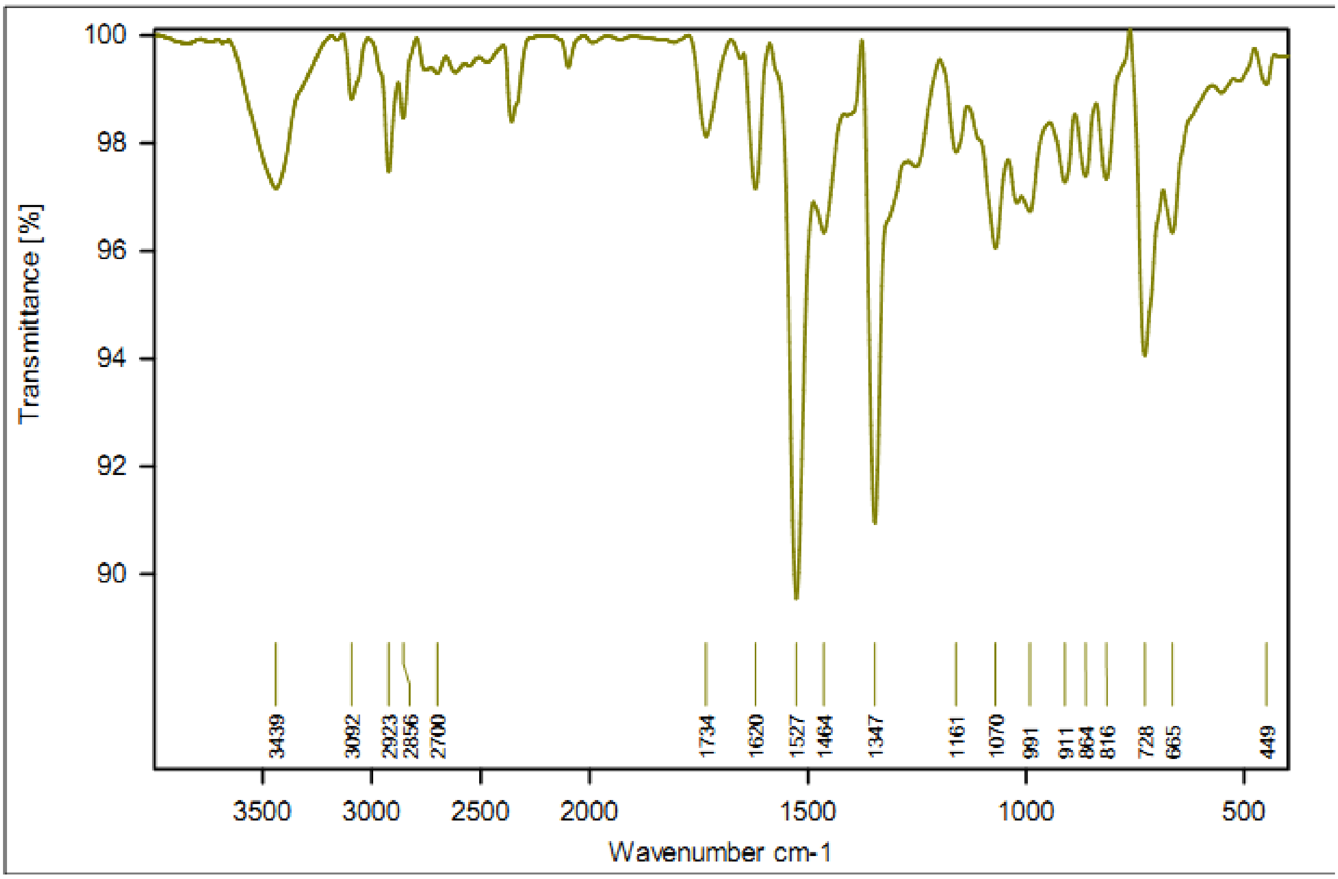

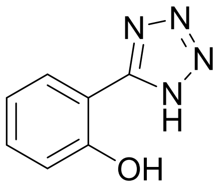

2-(1H-tetrazol-5-yl)phenol: ^{1}H NMR (400 MHz, DMSO-d6): δ_H= 7.99-7.96 (d of d, *J*= 12 Hz, *J*= 4 Hz, 1H), 7.42-7.37 (t of d, *J*= 12 Hz, 1H), 7.07-7.04 (d, *J*= 12 Hz, 1H), 7.02-6.96 (t, *J*= 12 Hz, 1H) ppm.

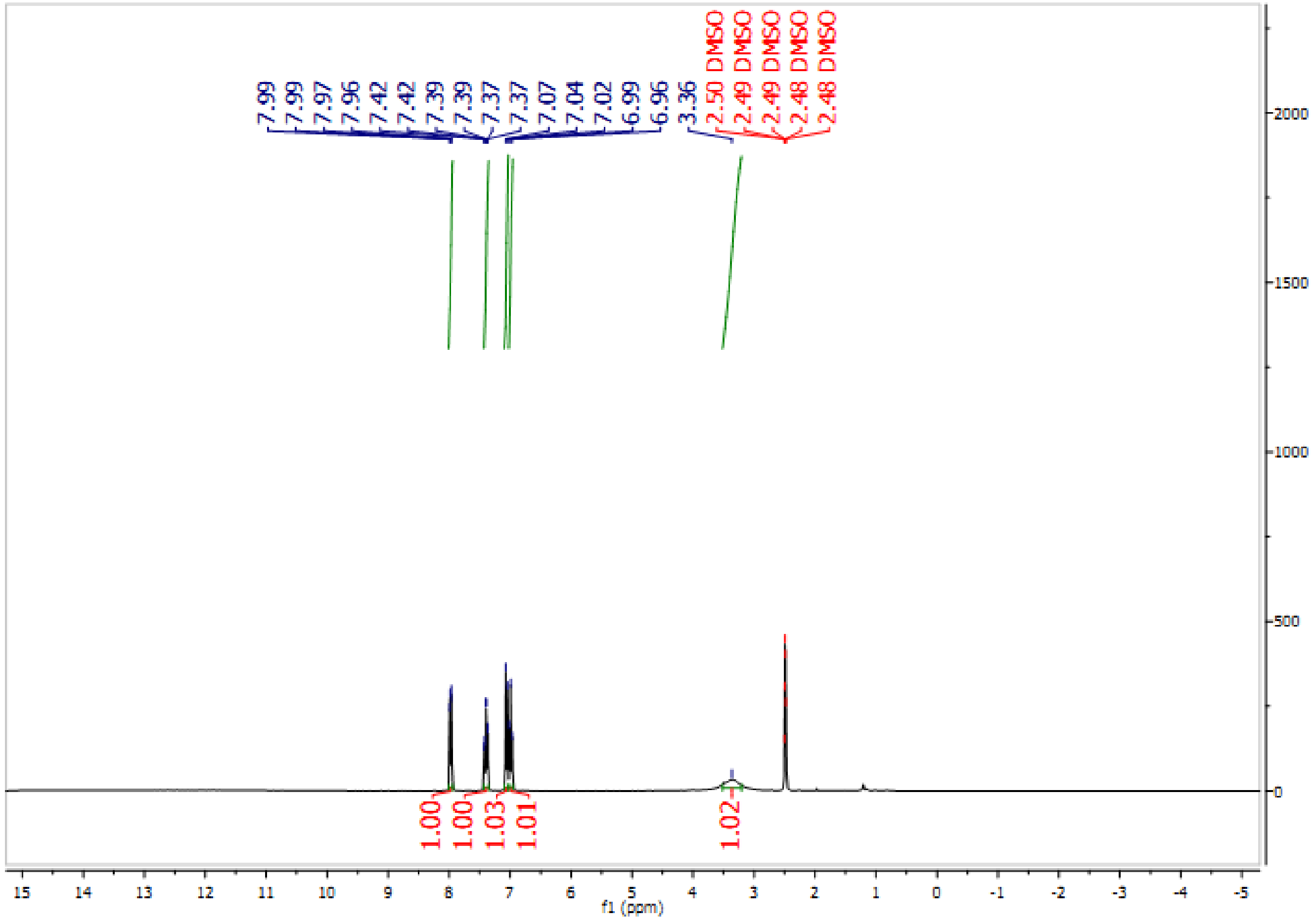

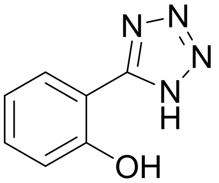

2-(1H-tetrazol-5-yl)phenol: ^{1}H NMR (400 MHz, DMSO-d6): δ_H= 7.99-7.96 (d of d, *J*= 12 Hz, *J*= 4 Hz, 1H), 7.42-7.37 (t of d, *J*= 12 Hz, 1H), 7.07-7.04 (d, *J*= 12 Hz, 1H), 7.02-6.96 (t, *J*= 12 Hz, 1H) ppm. ^{13}C NMR (400 MHz, DMSO-d6): δ_C= 155.3, 151.8, 132.5, 128.9, 119.7, 116.3, 110.6 ppm.

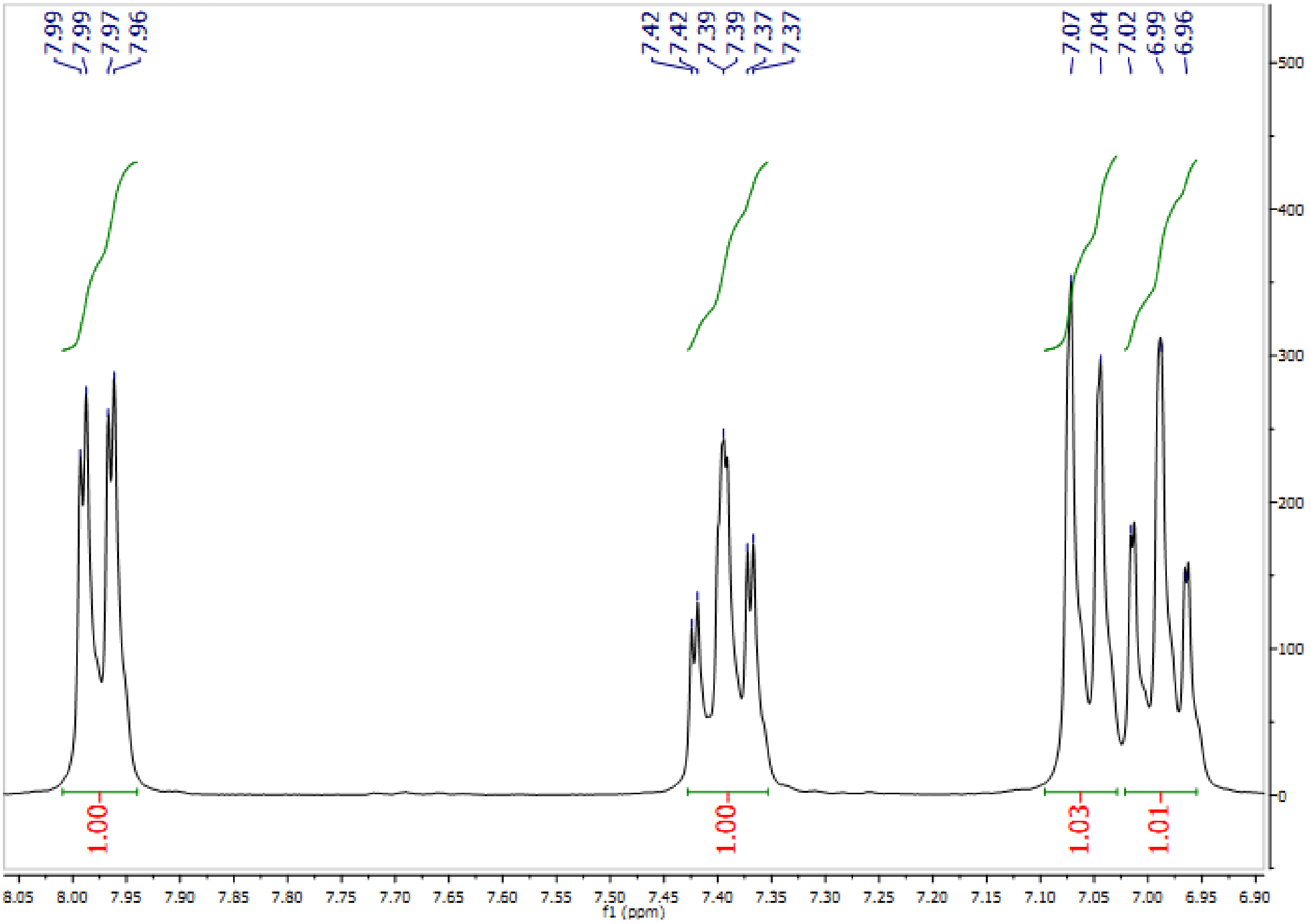

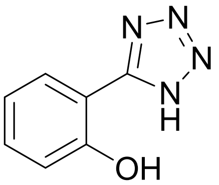

2-(1H-tetrazol-5-yl)phenol: ^{13}C NMR (400 MHz, DMSO-d6): δ_C= 155.3, 151.8, 132.5, 128.9, 119.7, 116.3, 110.6 ppm.

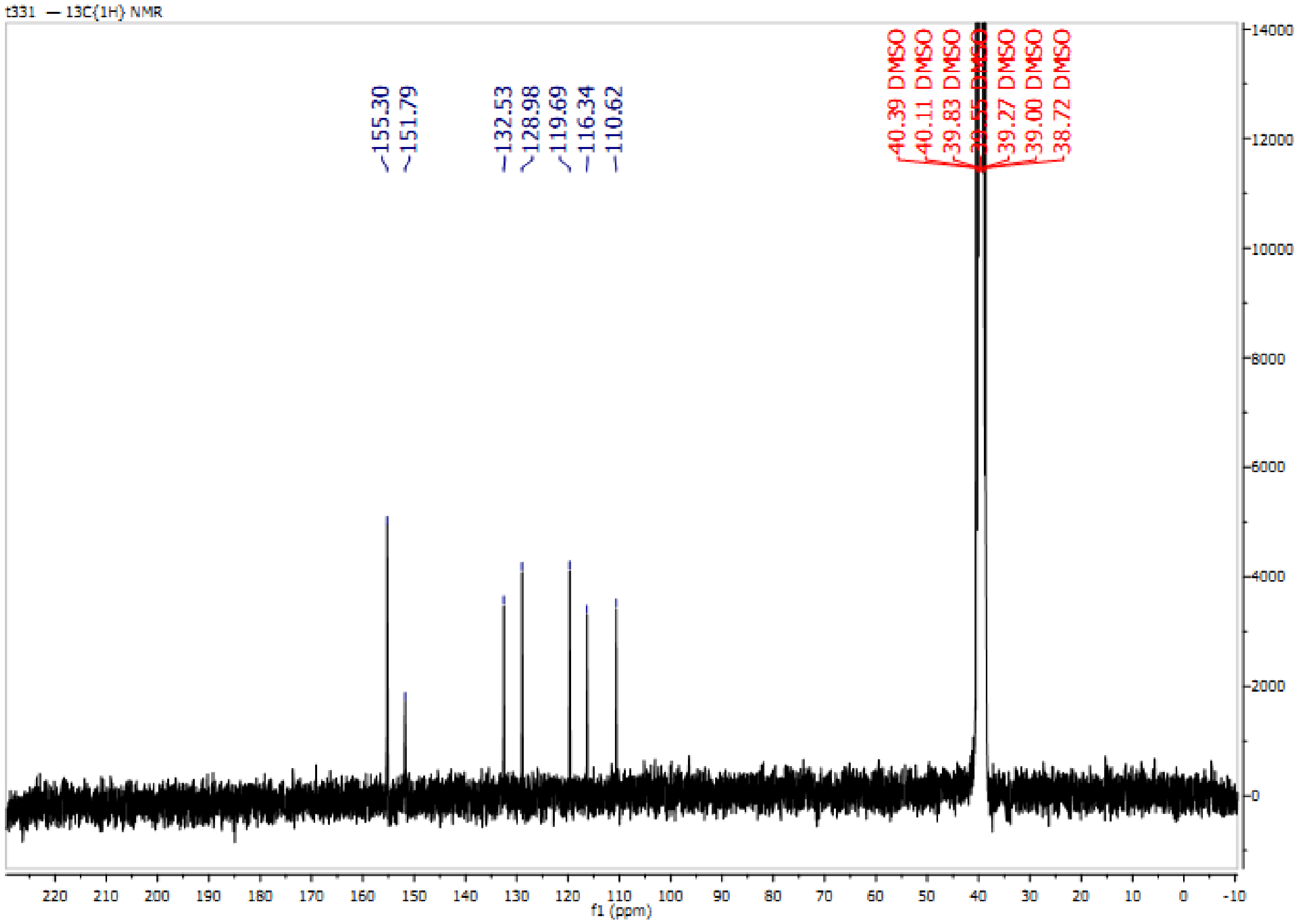

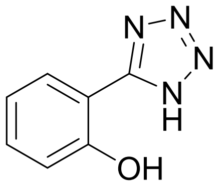

2-(1H-tetrazol-5-yl)phenol: IR (KBr) cm^{-1}:3253, 3058, 2941, 2708, 2565, 1892, 1735, 1610, 1546, 1476, 1393, 1358, 1294, 1230, 1150, 1114, 1067, 808, 742, 681, 538, 465.

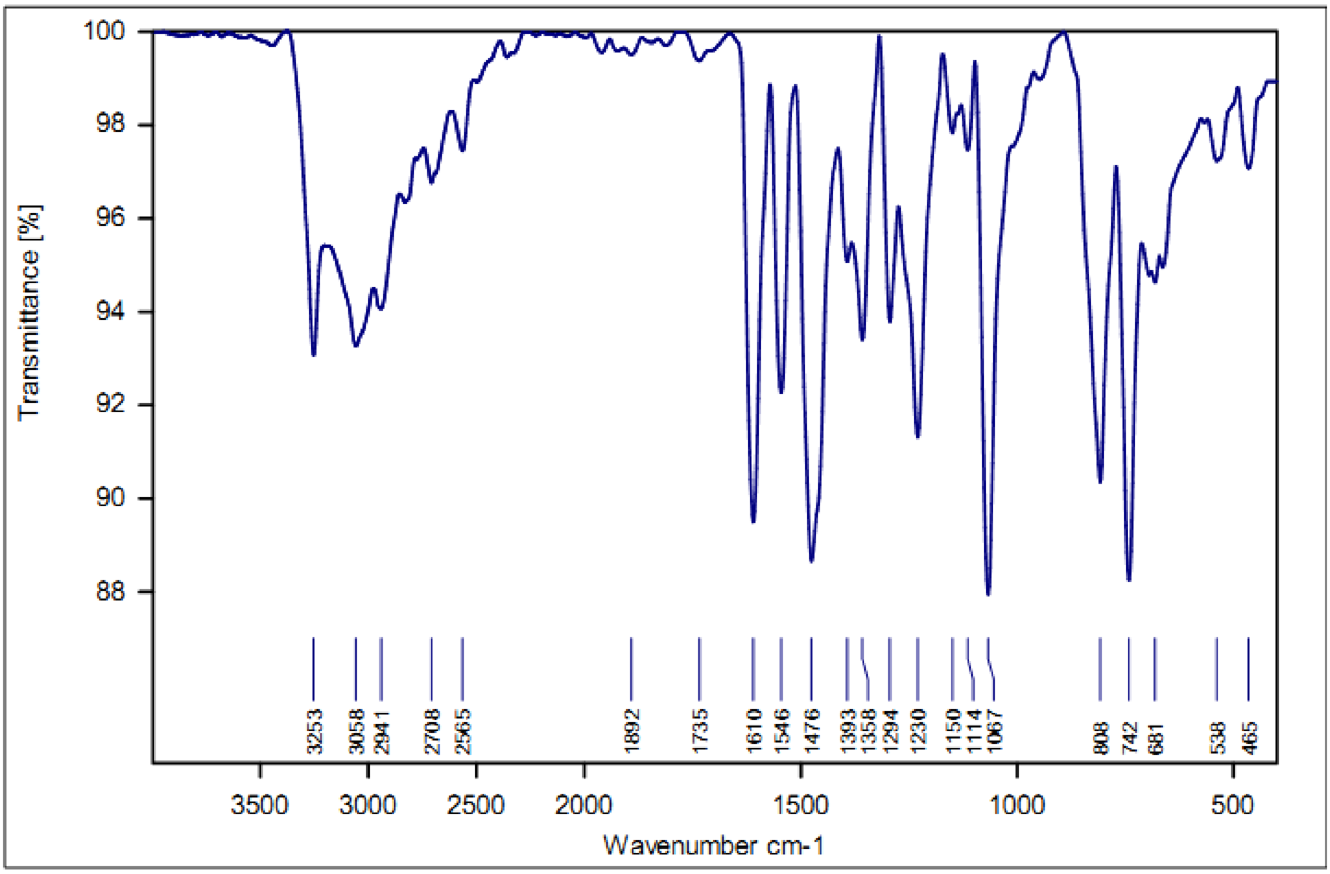

Supplement: RA-013-D2RA07510E-s001 [file RA-013-D2RA07510E-s001.pdf]
